# Iron and Copper Homeostasis in Cardiometabolic Disease: Therapeutic Potential of Chelators

**DOI:** 10.3390/ph19030441

**Published:** 2026-03-09

**Authors:** Joanna Izabela Lachowicz, Paweł Gać

**Affiliations:** Department of Environmental Health, Occupational Medicine and Epidemiology, Wroclaw Medical University, Mikulicza-Radeckiego 7, 50-368 Wroclaw, Poland; pawel.gac@umw.edu.pl

**Keywords:** ferroptosis, cuproptosis, cardiometabolic disease, iron chelation, copper homeostasis, oxidative stress, precision medicine

## Abstract

Cardiometabolic diseases remain a leading global health burden, and growing evidence indicates that dysregulation of iron and copper homeostasis plays a central role in their pathogenesis. Two metal-dependent forms of regulated cell death—ferroptosis and cuproptosis—have recently emerged as key mechanisms linking redox imbalance, mitochondrial dysfunction, vascular injury, and metabolic deterioration. This review synthesizes current mechanistic knowledge on iron- and copper-mediated cell death, with emphasis on their convergence at shared metabolic vulnerabilities, including glutathione depletion, instability of iron–sulfur clusters, and tricarboxylic acid cycle dependence. We integrate insights from single-cell transcriptomics, lipidomics, and metallomics with machine-learning-derived gene signatures to highlight novel biomarkers and vulnerability nodes relevant to coronary artery disease, myocardial infarction, heart failure, atherosclerosis, and diabetic complications. Special focus is placed on the therapeutic potential of metal chelators and metal-targeting pharmacological strategies, including mitochondria-directed copper depletion, iron chelation, radical-trapping antioxidants, copper ionophores, and dual-action approaches capable of rebalancing both metals simultaneously. Innovative delivery systems, such as targeted nanocarriers and copper-modulating microbubbles, are discussed in the context of precision redox medicine. Despite rapid progress, translation remains limited by biomarker variability, systemic safety concerns, and the lack of large, prospective clinical trials. Overall, the review positions the iron–copper axis as a mechanistically unified and therapeutically tractable target, offering new perspectives for the development of chelators and metal complexes in cardiometabolic disease management.

## 1. Introduction

Cardiometabolic diseases (CMDs)—including atherosclerosis, coronary artery disease (CAD), myocardial infarction (MI), heart failure (HF), and diabetes-related cardiac remodeling—remain the leading contributors to global morbidity and mortality despite major advances in prevention and therapy. Even under intensive risk-factor control with contemporary lipid-lowering, antihypertensive, antiplatelet, and glucose-lowering regimens, a substantial residual cardiovascular risk persists, particularly in people living with diabetes and metabolic syndrome (MetS). This persistent burden has sharpened attention on pathogenic mechanisms that operate downstream of classical risk factors, with a growing emphasis on redox biology, mitochondrial dysfunction, and trace-metal dyshomeostasis as convergent drivers of vascular and myocardial injury [1,2,3].

Over the last decade, oxidative stress has been reframed from a simple “oxidant–antioxidant imbalance” into a failure of a multilayered redox network that integrates metabolism, immunity, organellar crosstalk, and metal handling. Within this framework, two metal-dependent forms of regulated cell death (RCD) have emerged as central, actionable mechanisms in cardiometabolic pathology: ferroptosis and cuproptosis. Ferroptosis is an iron-driven programmed cell death characterized by glutathione (GSH) depletion, glutathione peroxidase-4 (GPX4) insufficiency, expansion of the labile iron pool, and polyunsaturated phospholipid peroxidation; it culminates in membrane failure, mitochondrial distress, and pro-inflammatory lipid signaling [2]. Cuproptosis, in contrast, is a copper-dependent program executed when mitochondrial Ferredoxin-1 (FDX1) reduces Cu^2+^ to Cu^+^, enabling aberrant binding to lipoylated tricarboxylic acid (TCA) cycle enzymes (e.g., Dihydrolipoamide Acetyltransferase (DLAT), Dihydrolipoamide Succinyltransferase (DLST), Dihydrolipoamide Branched-Chain Transacylase E2 (DBT), Glycine Cleavage System H-Protein (GCSH)), which induces proteotoxic aggregation, iron–sulfur (Fe–S) cluster loss, and collapse of oxidative phosphorylation [4,5].

**Scheme 1:**
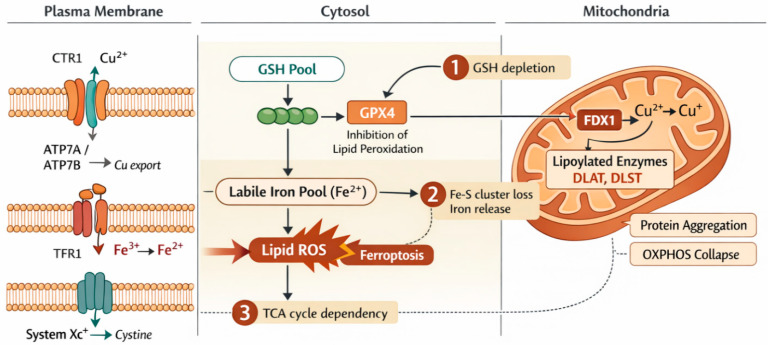
Crosstalk between cuproptosis and ferroptosis. The schematic illustrates the interplay between copper-induced cell death (cuproptosis) and iron-dependent cell death (ferroptosis) across cellular compartments. In the plasma membrane, copper uptake occurs via CTR1, while ATP7A/ATP7B mediate copper export. Iron is imported through transferrin receptor 1 (TfR1), and cystine uptake via System Xc^−^ supports GSH synthesis. In the cytosol, GSH maintains redox homeostasis and supports GPX4 activity, which inhibits lipid peroxidation. GSH depletion (Step 1) impairs GPX4, promoting lipid ROS accumulation and ferroptosis. The labile iron pool (Fe^2+^) contributes to ROS generation, while Fe-S cluster loss (Step 2) releases iron, further amplifying oxidative damage. In the mitochondria, copper binds to lipoylated enzymes (DLAT, DLST) via ferredoxin 1 (FDX1), triggering protein aggregation and collapse of oxidative phosphorylation (OXPHOS). This process is linked to TCA cycle dependency (Step 3), highlighting metabolic convergence between cuproptosis and ferroptosis.

Although ferroptosis and cuproptosis are biochemically distinct, they intersect at multiple nodes (Scheme 1) that are particularly relevant to cardiometabolic tissues. First, GSH depletion simultaneously sensitizes to ferroptosis (via GPX4 failure) and reduces intracellular copper buffering, thereby heightening cuproptotic susceptibility. Second, Fe–S cluster instability constitutes a bidirectional bridge: copper overload destabilizes Fe–S proteins, causing mitochondrial Fe^2+^ leakage and amplifying lipid peroxidation; conversely, disordered iron handling perturbs copper redox cycling [2,5]. Third, both programs show TCA/respiratory dependency: mitochondrial glutaminolysis potentiates ferroptosis, while the lipoylated TCA proteome is the executional target of cuproptosis; respiratory inhibition blunts both in preclinical systems [2]. Downstream, ferroptotic oxidized lipids and cuproptotic proteotoxic aggregates converge on innate immune activation and vascular remodeling, linking metabolic stress to chronic inflammation [5].

Compelling disease-level evidence now situates these pathways across the cardiometabolic continuum. In atherosclerosis, iron-driven lipid peroxidation destabilizes plaques, while cuproptosis-related genes (FDX1, Gene encoding Copper Transporter 1 (CTR1): SLC31A1/CTR1, Glutaminase (GLS)) are detectable in vascular datasets and associate with immune infiltration, suggesting a role for mitochondrial copper biology in lesion progression [2]. Hypertension exhibits ferroptotic remodeling via Angiotensin II—Interleukin-6/Signal Transducer and Activator of Transcription 3 (AngII–IL-6/STAT3) suppression of GPX4 and shows cuproptosis links through copper transport and efflux pathways (Sirtuin 7/Copper-transporting ATPases 7A; SIRT7/ATP7A) [2]. In heart failure, mitochondrial centrality dominates: iron overload drives mitochondrial ferroptosis, whereas the FDX1–lipoylation axis represents a putative cuproptosis target; chelators and mitochondria-supportive strategies may restore bioenergetics [2]. MI exemplifies copper’s “double edge”: adequate copper is pro-angiogenic through Hypoxia-Inducible Factor 1-Alpha/Vascular Endothelial Growth Factor (HIF-1α/VEGF), yet copper excess or mis-compartmentalization triggers Reactive Oxygen Species (ROS) generation, Fe–S destabilization, and cuproptosis, worsening injury and remodeling [4].

At the same time, single-cell and systems-biology approaches have deepened mechanistic resolution and opened translational avenues. Single-cell transcriptomics across vascular and cardiac tissues reveals cell-type-specific expression of ferroptosis (e.g., Solute Carrier Family 7 Member 11 (SLC7A11), GPX4, Acyl-CoA Synthetase Long-Chain Family Member 4 (ACSL4), Nuclear Receptor Coactivator 4 (NCOA4), Ferroptosis Suppressor Protein 1 (FSP1), Dihydroorotate Dehydrogenase (DHODH)) and cuproptosis programs (e.g., SLC31A1/CTR1, Antioxidant 1 Copper Chaperone (ATOX1), ATP7A/B, FDX1, DLAT/DLST/DBT/GCSH), while integrative bioinformatics in CAD has identified composite gene signatures that merge cuproptosis, ferroptosis, and pyroptosis (CFP) biology. In particular, Weighted Gene Co-expression Network Analysis (WGCNA) combined with machine learning has nominated nine CAD classifiers—C-terminal Domain (CTD) Small Phosphatase 2 (CTDSP2), Dehydrogenase/Reductase 7 (DHRS7), NOD-Like Receptor Pyrin Domain Containing 1 (NLRP1), Myristoylated Alanine-Rich Protein Kinase C Substrate (MARCKS), Pellino E3 Ubiquitin Protein Ligase 1 (PELI1), Rab-Interacting Lysosomal Protein-Like 2 (RILPL2), JunB Proto-Oncogene, AP-1 Transcription Factor Subunit (JUNB), Serine/Threonine Kinase 17B (STK17B), Gene encoding Ferroportin (SLC40A1)—with excellent discriminative performance and experimental validation of STK17B in endothelial migration/invasion, strengthening causal plausibility [6]. These developments align with the concept of “redox phenotyping”, wherein redox lipidomics (oxidized phospholipid panels), metallomics (labile Fe/Cu pools), and gene signatures are integrated—potentially with Artificial Intelligence (AI)—to stratify patients and guide therapy [5].

Therapeutically, the recognition that ferroptosis and cuproptosis are metal-gated, mitochondria-centered suggests a unifying strategy: rebalance metals, protect membranes, and preserve mitochondrial function. Ferroptosis mitigation includes radical-trapping antioxidants (e.g., ferrostatin-1, Lip-1-1), iron chelation (deferoxamine (DFO), deferiprone (DFP), deferasirox (DFX)), GPX4/FSP1/DHODH support, and Nuclear Factor Erythroid 2–related Factor 2 (NRF2) activation; cuproptosis modulation employs copper chelators (trientine, tetrathiomolybdate) for overload, ionophores (elesclomol) for controlled delivery in deficiency contexts, and FDX1/lipoylation targeting for specificity [2,4]. Encouragingly, nanomedicine and targeted delivery platforms—such as copper-depleting/ceria hybrids or ultrasound-guided copper microbubbles—offer compartment-specific control that could improve benefit–risk profiles in ischemic myocardium and diseased vessels [4].

Despite this momentum, translation remains early. Many biomarkers are research-grade with limited standardization; serum copper signals and Cu:Zn ratios vary across cohorts; and large, biomarker-guided trials testing dual-pathway modulation (iron with copper) are only beginning to be conceptualized. Safety is a parallel priority: both chelators and ionophores can disrupt essential metal balance, and compartment-specific targeting will be critical to minimize off-target toxicity [4,5].

In light of these developments, the present review focused on iron–copper crosstalk is timely. Here, we (i) consolidate mechanistic evidence for ferroptosis and cuproptosis as intertwined drivers of CMDs; (ii) integrate single-cell and multi-omic signatures that map cell-type-specific vulnerability; (iii) evaluate diagnostic opportunities in “redox phenotyping” using lipidomics, metallomics, and CFP gene panels; and (iv) appraise therapeutic strategies spanning chelation, antioxidants, metabolic support, and targeted delivery—with a particular emphasis on dual-action approaches that co-modulate iron and copper. We also articulate limitations in assay specificity, phenotyping, and clinical evidence, and we outline future directions—including compartment-specific interventions, AI-guided stratification, and rigorously designed, biomarker-anchored trials—to translate metal-targeted strategies into precision redox cardiology.

### Literature Selection Methodology

We employed a structured and transparent search strategy to ensure comprehensive coverage and reproducibility. Literature was identified through searches conducted between January 2010 and January 2026 in major medical and scientific databases, including PubMed, Scopus, Web of Science, and Google Scholar. Additional relevant clinical trial information was obtained through ClinicalTrials.gov. The primary search terms included combinations of: “ferroptosis”, “cuproptosis”, “regulated cell death”, “iron overload”, “copper homeostasis”, “iron chelation”, “copper chelation”, “cardiometabolic disease”, “atherosclerosis”, “myocardial infarction”, “heart failure”, “diabetes”, and “diabetic cardiomyopathy”. Targeted terms related to therapeutic strategies included “chelation therapy”, “nanocarriers”, “nanoparticles”, “mitochondrial targeting”, and “metal dyshomeostasis”. Reference lists of key papers and recent reviews were screened manually to identify additional relevant studies. Inclusion Criteria: (i) Peer-reviewed original research articles, reviews, meta-analyses, and clinical trials. (ii) Studies investigating iron or copper dysregulation, ferroptosis, cuproptosis, or associated pathways in cardiometabolic diseases. (iii) Experimental (in vitro, in vivo), translational (human tissue or multi-omics), and clinical studies reporting mechanistic or therapeutic insights. Exclusion Criteria: (i) Articles not related to cardiometabolic diseases or metal-dependent pathways. (ii) Studies lacking mechanistic or therapeutic relevance. (iii) Non-English publications. Both authors independently reviewed full texts to ensure relevance and consistency. The search strategy prioritized mechanistic, translational, and therapeutic evidence, with special emphasis on studies examining chelation therapy, metal-targeted modulation, and regulated cell death pathways in cardiometabolic contexts.

## 2. Role of Iron and Ferroptosis in Cardiometabolic Diseases

Iron is an indispensable trace element required for fundamental biological processes, including oxygen transport, DNA synthesis, and energy metabolism [7]. Under physiological conditions, iron homeostasis is tightly regulated through coordinated control of absorption, transport, and storage by proteins such as transferrin, ferroportin, and the hormone hepcidin. This regulation is critical because humans lack an efficient mechanism for iron excretion, making both deficiency and overload clinically significant [8].

*(a)*  *Mechanistic role of iron in cardiovascular diseases (CVDs)*

**Scheme 2:**
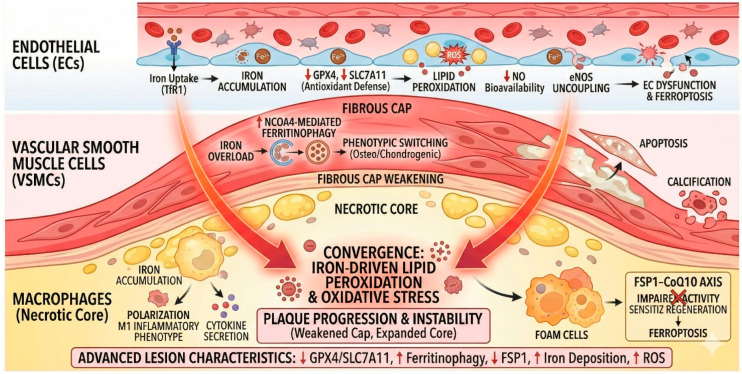
Ferroptotic mechanisms in atherosclerotic plaque progression. The schematic illustrates the role of ferroptosis in the development and destabilization of atherosclerotic lesions. In ECs, iron uptake via TFR1 leads to iron accumulation, reduced antioxidant defense (↓GPX4, ↓SLC7A11), and increased lipid peroxidation, contributing to endothelial dysfunction and ferroptosis. Loss of nitric oxide (NO) bioavailability and eNOS uncoupling further exacerbate oxidative stress. In VSMCs, NCOA4-mediated ferritinophagy promotes iron release, driving phenotypic switching toward osteo/chondrogenic states, which weakens the fibrous cap and predisposes to calcification and apoptosis. In the necrotic core, macrophages exhibit iron accumulation, polarization toward an M1 inflammatory phenotype, and cytokine secretion, amplifying oxidative stress. The convergence of iron-driven lipid peroxidation and ROS generation accelerates plaque progression and instability, characterized by fibrous cap weakening and necrotic core expansion. Advanced lesion features include decreased GPX4/SLC7A11 expression, increased ferritinophagy, iron deposition, and elevated ROS. Impairment of the FSP1-CoQ10 axis further sensitizes cells to ferroptosis, highlighting its critical role in atherogenesis.

When iron balance is disrupted, profound metabolic and vascular consequences ensue. Iron overload, particularly in the form of non-transferrin-bound iron (NTBI), has emerged as a key contributor to atherosclerosis, a leading cause of cardiovascular morbidity and mortality. Excess iron catalyzes Fenton and Haber–Weiss reactions, generating ROS that oxidize lipids, proteins, and nucleic acids. This oxidative milieu accelerates endothelial dysfunction, promotes vascular smooth muscle cell (VSMC) calcification, and enhances macrophage foam cell formation (Scheme 2)—hallmarks of plaque progression and instability [9,10,11].

Beyond atherosclerosis, iron dysregulation intersects with metabolic disease. Recent evidence links iron overload to type 2 diabetes mellitus (T2DM)**,** where elevated systemic and tissue iron levels correlate with insulin resistance, β-cell dysfunction, and chronic complications. Mechanistically, iron-driven oxidative stress impairs insulin signaling and exacerbates inflammatory pathways, creating a vicious cycle of metabolic deterioration [12,13,14,15,16,17,18]. These findings underscore iron’s dual role as an essential micronutrient and a potential pathogenic factor when homeostasis fails.

Iron is indispensable for oxygen carriage (hemoglobin, myoglobin), electron transfer within the mitochondrial respiratory chain, and numerous oxidoreductase reactions. Because humans lack an efficient excretory pathway for iron, systemic balance hinges on tightly coordinated absorption, transport, storage, and recycling—principally via duodenal uptake, transferrin-mediated delivery, macrophage recycling, and the hepcidin–ferroportin axis that restricts egress of cellular iron when stores are sufficient. Perturbations at any of these checkpoints reverberate across cardiovascular and metabolic tissues, with both deficiency and overload exerting clinically meaningful effects [19].

Iron deficiency is common in HF and pulmonary arterial hypertension (PAH) and presents along two mechanistic lines. Absolute deficiency reflects depleted stores from inadequate intake, malabsorption, or chronic blood loss; functional deficiency reflects iron sequestration during chronic inflammation, in which hepcidin-driven ferroportin blockade limits bioavailability despite normal or elevated ferritin. Irrespective of phenotype, iron deficiency compromises hemoglobin and myoglobin function, lowers oxygen delivery to metabolically active myocardium and skeletal muscle, and manifests clinically as reduced exercise capacity, tissue hypoxia, and heightened hospitalization risk. Randomized trials in HF demonstrate that intravenous ferric carboxymaltose ameliorates symptoms and health status—even in non-anemic patients—underscoring the importance of iron beyond erythropoiesis [8]. In the large FAIR-HF2 randomized trial, ferric carboxymaltose did not significantly reduce cardiovascular death or heart failure hospitalizations but it consistently improved patient-reported quality of life and was well tolerated, with similar rates of serious adverse events to placebo. In HFpEF, the smaller FAIR-HFpEF trial—terminated early—showed that ferric carboxymaltose improved functional capacity, with a significant increase in 6 min walk distance and fewer serious adverse events; however, effects on symptoms and quality of life could not be confirmed due to limited power [20].

A 2026 meta-analysis of nine trials including over 6400 patients demonstrated that ferric carboxymaltose produced a modest but statistically significant reduction in the composite of heart failure hospitalization or cardiovascular mortality, alongside improvements in exercise capacity, though no mortality benefit was observed. Subgroup analyses indicate that benefits are generally consistent across ischemic and non-ischemic HF etiologies, supporting broad therapeutic applicability [21].

At the opposite extreme, iron overload—arising from hereditary hemochromatosis, transfusional siderosis, or excessive supplementation—drives myocardial iron deposition, catalyzes oxidative reactions, and disables mitochondrial bioenergetics. Federti et al. [22] showed that Nrf2 is essential for protecting the heart from chronic oxidative and iron-induced injury. Mice lacking Nrf2 develop age-dependent cardiomyopathy characterized by severe oxidative stress, SERCA2a degradation, inflammation, and fibrosis, worsened by local cardiac iron accumulation. Under dietary iron overload, Nrf2-deficient mice paradoxically avoid further cardiac iron loading because strong activation of the unfolded protein response (UPR) induces cardiac hepcidin, diverting iron to the liver—but this comes at the cost of greater ER stress, apoptosis, and hypertrophy. In β-thalassemia mice, cardiomyopathy arises mainly from persistent oxidative and proteotoxic stress, even without measurable cardiac iron, again involving UPR activation, SERCA2a loss, and fibrosis.

Vascularly, iron accumulation in endothelial cells (ECs) leads to ferroptotic loss of nitric-oxide bioavailability, Endothelial Nitric Oxide Synthase (eNOS) uncoupling, and pro-inflammatory signaling. In VSMCs, iron excess drives phenotypic switching toward osteo/chondrogenic programs, fosters apoptosis, and promotes neointimal hyperplasia and calcification (Scheme 2). Iron-loaded macrophages skew toward an M1 inflammatory phenotype, augment glycolysis, secrete cytokines, and form lipid-laden foam cells—all processes that weaken the fibrous cap and enlarge the necrotic core [19]. These cellular events scale to the tissue level across all stages of atherogenesis: initiation (EC dysfunction and leukocyte recruitment), progression (foam cell expansion, efferocytosis failure, necrotic core growth), and advanced disease (cap thinning, intraplaque hemorrhage, plaque rupture). Mechanistically, ferritinophagy (NCOA4-mediated), accumulation of PUFA-rich phospholipids, and ROS propagation are central drivers, while GPX4 and the FSP-1-Coenzyme Q10 (FSP1–CoQ10) axis serve as endogenous brakes. Advanced lesions typically show depressed GPX4/SLC7A11, heightened lipid peroxidation, and iron deposition; intraplaque hemorrhage further enlarges the labile iron pool via heme catabolism, potentiating ferroptosis and structural failure [23].

Crosstalk with autophagy (via ferritinophagy) [24], pyroptosis, and necroptosis is increasingly recognized [25], and metabolic stressors characteristic of cardiometabolic disease—hyperglycemia, IR, and chronic inflammation—create a permissive microenvironment for ferroptotic execution in cardiomyocytes, ECs, VSMCs, and immune cells [24,25].

*(b)*  *Mechanistic role of iron in diabetes*

Emerging evidence implicates ferroptosis as a key contributor to the pathophysiology of diabetes and its complications [26] (Table 1). In pancreatic β cells, ferroptotic stress impairs viability and insulin secretory capacity. In insulin-responsive tissues—hepatocytes, adipocytes, and skeletal muscle—iron-driven oxidative lipid damage compromises insulin signaling and substrate utilization, collectively promoting hyperglycemia and insulin resistance [23,26].

**Scheme 3:**
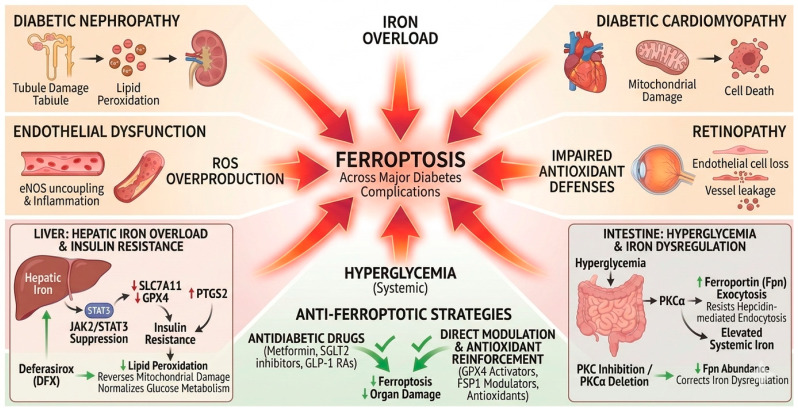
Ferroptosis in diabetes complications and associated mechanisms. The schematic summarizes the contribution of ferroptosis to major diabetes-related complications. Central to the diagram is ferroptosis, driven by systemic iron overload, hyperglycemia, and impaired antioxidant defenses. In DN, tubular damage and lipid peroxidation promote renal dysfunction. Endothelial dysfunction arises from eNOS uncoupling and excessive ROS production, leading to inflammation. Diabetic Cardiomyopathy (DiaCM) involves mitochondrial damage and ferroptotic cell death, while retinopathy is characterized by endothelial cell loss and vessel leakage. In the liver, hepatic iron overload and insulin resistance are linked to decreased SLC7A11 and GPX4 expression, increased PTGS2 activity, and JAK2/STAT3 suppression, which exacerbate lipid peroxidation and metabolic dysregulation. In the intestine, hyperglycemia and iron dysregulation impair Fpn exocytosis, increase hepcidin-mediated iron retention, and elevate systemic iron levels; PKCα inhibition or deletion further alters iron homeostasis. Anti-ferroptotic strategies include antidiabetic drugs (metformin, SGLT2 inhibitors, GLP-1 receptor agonists) and direct modulation of antioxidant systems (GPX4 activators, FSP1 modulators, and exogenous antioxidants), aiming to reduce ferroptosis and prevent organ damage.

Ferroptosis is also implicated in major diabetes-related complications (Scheme 3), including diabetic nephropathy (DN), cardiomyopathy, retinopathy, and endothelial dysfunction, through convergent mechanisms involving iron overload, ROS overproduction, and impaired antioxidant defenses.

A recent extensive overview by Jin et al. [14,26] underscores the central role of iron metabolism and ferroptosis in diabetes pathophysiology. Pancreatic β cells, endowed with relatively weak antioxidant defenses, are highly susceptible to ferroptotic injury, leading to β-cell loss and impaired glucose-stimulated insulin secretion; pharmacological inhibition with ferroptosis blockers (e.g., Fer-1) preserves β-cell viability. Iron overload exacerbates oxidative stress through Fenton chemistry, driving ROS accumulation and lipid peroxidation in β cells, hepatocytes, and adipocytes, thereby propagating insulin resistance and meta-inflammation [27]. Ferroptosis is implicated across diabetic complications, including nephropathy (podocyte injury and tubular cell death), cardiomyopathy (fibrotic remodeling and mitochondrial dysfunction), retinopathy, and endothelial dysfunction. Mechanistic links involve dysregulated iron homeostasis, suppression of nuclear factor erythroid 2–related factor 2 (NRF2)-dependent antioxidant responses [28], and activation of ACSL4 [28] and SLC7A11 [27].

A systematic review of 21 studies reported consistent elevations in serum ferritin among non-hemochromatosis diabetic patients (mean +27.6%, median +11.9%), correlating with microvascular complication risk [29]. Proposed mechanisms include hepcidin upregulation and hemolysis-driven ferritin increases via Glycated Hemoglobin A1c (HbA1c)-related erythrocyte osmotic fragility. Elevated ferritin is associated with insulin resistance through ROS-induced mitochondrial fission, while apoferritin may impair Glucose Transporter Type 4 (GLUT4) translocation by activating PI3K–3-Phosphoinositide-Dependent Protein Kinase-1 (PDK1) pathways.

Mo et al. [27] investigated the causal link between hepatic iron excess and insulin resistance, focusing on ferroptosis as the effector mechanism. In complementary in vivo and in vitro models, iron overload increased oxidative stress, depleted GSH, suppressed GPX4, and induced mitochondrial injury, concomitant with impaired glucose tolerance and reduced insulin sensitivity. Mechanistically, iron excess suppressed phosphorylation of Janus Kinase 2 JAK2/STAT3 and downregulated SLC7A11 and GPX4, while inducing Prostaglandin-Endoperoxide Synthase 2 (PTGS2) expression—molecular changes consistent with ferroptotic commitment.

Banerjee et al. [30] delineated how hyperglycemia-activated protein kinase C alpha (PKCα) modulates the iron exporter ferroportin (Fpn) to promote diabetic iron overload. Across type 1 and type 2 diabetic mouse models and human duodenal biopsies, hyperglycemia enhanced PKCα-dependent trafficking of Fpn to the plasma membrane and inhibited its hepcidin-mediated endocytosis and degradation, resulting in persistent iron export from enterocytes and elevated systemic iron. Direct PKCα–Fpn interaction favored Fpn exocytosis and prevented endocytic retrieval, overriding canonical iron-sensing feedback.

Iron excess is increasingly recognized as a proximal driver of β-cell failure in type 2 diabetes. In C57BL/6J mice and MIN6 β cells, Deng et al. [31] showed that iron overload impairs insulin secretion and disrupts glucose homeostasis through ferroptosis. Ultrastructural analyses demonstrated mitochondrial shrinkage, loss of cristae, and endoplasmic reticulum swelling, while biochemical assays confirmed elevated ROS and malondialdehyde (MDA). At the signaling level, iron overload activated the Apoptosis Signal-Regulating Kinase 1/Stress-Activated Mitogen-Activated Protein Kinase/C/EBP Homologous Protein (ASK1/p38/CHOP) stress axis, linking oxidative stress to endoplasmic reticulum (ER) stress and ferroptosis.

Chen & Zhang [32] examined the NCOA4–Ferritin Heavy Chain (FTH1) axis in DN, focusing on ferritinophagy-mediated iron mobilization. In db/db mice and renal cells exposed to high-glucose/high-fat conditions, they observed increased ferrous iron, lipid peroxidation, and mitochondrial damage consistent with ferroptosis, accompanied by dysregulated expression of NCOA4 and FTH1. Functional perturbations revealed that FTH1 knockdown intensified ferroptosis and fibrotic remodeling, whereas NCOA4 silencing restored FTH1 levels and mitigated oxidative injury and interstitial fibrosis. Mechanistically, hypoxic/high-glucose conditions upregulated NCOA4 in a HIF-1α-dependent manner.

**Table 1:** Summary of the mechanistic roles of iron and ferroptosis in cardiometabolic diseases. This table synthesizes the key pathogenic mechanisms linking iron dysregulation and ferroptosis across cardiometabolic contexts, including atherosclerosis, heart failure, pulmonary hypertension, and diabetes-related complications. It outlines the major biological processes affected—such as oxidative stress, ferritinophagy, endothelial dysfunction, metabolic impairment, and regulated cell death—and summarizes supporting evidence from animal models, in vitro studies, human tissue analyses, clinical trials, and systematic reviews. Underlined study names correspond to seminal or pivotal investigations cited in the manuscript.

Topic/Mechanistic Area	Key Findings	Type of Study	Reference No.
Iron overload and atherosclerosis	NTBI promotes ROS, endothelial dysfunction, VSMC calcification, foam cell formation → plaque instability.	clinical observational human study; rabbit and mouse model	[9,10,11]
Iron overload and type 2 diabetes	High iron correlates with insulin resistance, β-cell dysfunction, oxidative stress, and inflammation.	animal models, cell culture experiments, interventional designs, and mechanistic laboratory work	[12,13,14,15,16,17,18]
Iron deficiency in HF and PAH	Reduces oxygen delivery and performance; IV ferric carboxymaltose improves symptoms.	Clinical trials: NCT01922479; NCT00520780	Clinicaltrial.gov
Iron overload in cardiomyopathy and vascular injury	Excess labile iron drives ROS, mitochondrial injury, arrhythmias; accelerates atherogenesis.	Mouse model	[22]
Ferritinophagy and plaque instability	NCOA4-induced ferritin breakdown increases labile iron → lipid peroxidation/structural failure.	In vitro cellular studies	[33]
Iron overload → hepatic insulin resistance	Iron excess suppresses JAK2/STAT3/SLC7A11; ↑PTGS2; DFX reverses glucose intolerance and lipid peroxidation.	Animal model + in vitro	[27]
PKCα regulation of ferroportin in diabetes	Hyperglycemia activates PKCα → ↑Fpn trafficking; blunts hepcidin endocytosis → systemic iron elevation.	Animal models + human tissue + in vitro	[30]
β-cell ferroptosis in T2DM	Iron overload damages mitochondria/ER; activates ASK1/p38/CHOP; DFX partially protective.	Animal model + in vitro	[31]
Ferritinophagy (NCOA4–FTH1) in DN	NCOA4 upregulation → Fe^2+^ release, lipid peroxidation, fibrosis; metformin/YC-1 suppress HIF1α–NCOA4 axis.	Animal model + in vitro	[32]

## 3. Iron Chelation in Cardiometabolic Diseases

In cases of iron deficiency, therapeutic approaches typically involve oral or intravenous iron supplementation combined with dietary interventions to restore systemic iron balance. A recent systematic review of clinical evidence [34] confirms that intravenous iron therapy significantly improves outcomes in patients with heart failure and iron deficiency. Conversely, iron overload is managed through chelation therapy using agents such as DFO, DFP, or DFX, alongside phlebotomy and emerging treatments with hepcidin analogs.

Ferroptosis is addressed experimentally through strategies that enhance GPX4 activity, inhibit ACSL4, modulate glutaminolysis, and scavenge mitochondrial reactive oxygen species using agents such as MitoTEMPO [8].

Therapeutically, ferroptosis is tractable. In preclinical systems, iron chelators, radical-trapping antioxidants, GPX4 activators, and ACSL4 inhibitors attenuate myocardial injury, improve endothelial function, and stabilize plaques by reducing lipid peroxidation and preserving mitochondrial function [8,30]. These interventions complement clinical strategies that correct deficiency (parenteral iron repletion in HF) or overload (chelation in hereditary or transfusional siderosis), collectively pointing to iron as both a biomarker and a modifiable therapeutic axis in cardiometabolic medicine.

Ferroptosis inhibition has demonstrated efficacy in reducing plaque progression and stabilizing atherosclerotic lesions in experimental models. Therapeutic modalities include ferroptosis inhibitors such as Ferrostatin-1 (Fer-1) and Liproxstatin-1 (Lip-1), iron chelators, and antioxidant strategies. Innovative approaches under investigation encompass microRNA delivery via endothelial progenitor cell-derived vesicles and dietary modulation of polyunsaturated and monounsaturated fatty acid ratios [23]. For CMDs, additional strategies involve lipoxygenase inhibitors, ACSL4 inhibitors, nitroxides, and selenium supplementation, aiming to restore iron homeostasis, suppress lipid peroxidation, and reinforce antioxidant defenses [35].

Targeting ferroptosis offers a novel avenue for T2DM management. Agents such as metformin, quercetin, melatonin, and vitamin D exhibit anti-ferroptotic activity by modulating key signaling pathways, including Adenosine Monophosphate (AMP)-activated protein kinase (AMPK) activation, NRF2 signaling, and Nuclear Factor kappa-light-chain-enhancer of activated B cells (NF-κB)/Divalent Metal Transporter 1 (DMT1) regulation. While preclinical data are promising, clinical trials remain absent, and the lack of validated ferroptosis biomarkers hampers translational progress.

*(a)*  *Iron chelation in CVDs*

Modulation of iron homeostasis represents a promising strategy for the management of atherosclerosis. Preclinical evidence supports the use of iron chelators, ferroptosis inhibitors, and antioxidant compounds to attenuate iron-driven oxidative stress and lipid peroxidation, thereby reducing plaque progression [19].

Atherosclerosis is a chronic inflammatory disease characterized by oxidative stress and upregulation of adhesion molecules such as VCAM-1 and ICAM-1, which promote monocyte recruitment and plaque initiation [36]. Lipopolysaccharide (LPS), a potent pro-inflammatory stimulus, activates NF-κB signaling and ROS generation. Nicotinamide Adenine Dinucleotide Phosphate (NADPH) oxidase is a major source of ROS in vascular cells, and its catalytic subunit p22^phox requires heme for structural stability, implicating iron in enzyme function. Excess iron may exacerbate oxidative stress, whereas iron chelation could attenuate inflammatory signaling.

In a murine air pouch model, Li and Frei [37] demonstrated that LPS markedly increased leukocyte infiltration, NADPH oxidase activity, ROS production, NF-κB activation, and VCAM-1/ICAM-1 expression. Treatment with DFO significantly inhibited p22^phox upregulation, blocked NADPH oxidase activity and ROS generation, and prevented NF-κB nuclear translocation. These effects were abolished by Fe(III)-loaded DFO, confirming iron chelation as the underlying mechanism. Adhesion molecule expression was strongly reduced by DFO and apocynin. These findings indicate that iron sustains NADPH oxidase-driven oxidative stress and NF-κB activation, promoting vascular inflammation, and highlight iron chelation as a potential therapeutic strategy for atherosclerotic vascular disease.

Anthracyclines such as doxorubicin (DOX) remain essential in pediatric oncology but are associated with a high risk of cardiotoxicity, primarily driven by iron-mediated ROS generation. DOX forms complexes with intracellular iron, catalyzing hydroxyl radical formation and oxidative damage to cardiomyocytes. Dexrazoxane is currently the only approved cardioprotective agent; however, alternative iron chelators such as DFO may offer similar benefits. In a randomized trial involving 62 pediatric cancer patients receiving DOX-based chemotherapy, Rahimi et al. [38] evaluated the cardioprotective effect of DFO administered intravenously at either 10× the DOX dose or 50 mg/kg around each infusion. No clinical cardiotoxicity or adverse effects were observed. NT-proBNP levels were significantly lower in the high-dose DFO group compared to controls (*p* = 0.036), suggesting molecular-level protection, while troponin I remained undetectable. Echocardiography revealed marginal improvement in ejection fraction and subtle diastolic changes in DFO-treated patients. These findings indicate that high-dose DFO may reduce subclinical cardiac injury during DOX therapy without toxicity. Larger, long-term studies are warranted to confirm efficacy and compare DFO with dexrazoxane for the prevention of chronic anthracycline cardiotoxicity.

Cardiac iron overload is a major determinant of morbidity and mortality in β-thalassemia major. The CORDELIA phase II trial (NCT00600938) assessed the non-inferiority of oral DFX compared with subcutaneous DFO for cardiac iron removal in patients with myocardial siderosis and preserved cardiac function [39]. A total of 197 patients (mean age ~19.8 years) with cardiac T_2_star values between 6 and 20 ms and left ventricular ejection fraction (LVEF) ≥ 56% were enrolled across 22 centers. Participants received either DFX (40 mg/kg/day) or DFO (50–60 mg/kg/day, 5–7 days/week) for 12 months. The primary endpoint was change in myocardial T_2_star assessed by cardiac Magnetic Resonance Imaging (MRI); secondary endpoints included LVEF stability and safety. Both treatments significantly improved T_2_star (DFX: 11.2 → 12.6 ms; DFO: 11.6 → 12.3 ms), with a geometric mean ratio of 1.056 (95% CI: 0.998–1.133), meeting non-inferiority criteria. LVEF remained stable in both groups, and adverse event rates were comparable (DFX: 35.4%; DFO: 30.8%), with no unexpected safety signals. These findings confirm that DFX is as effective and safe as DFO for cardiac iron removal, offering the advantage of oral administration and improved treatment convenience. Long-term studies are needed to evaluate outcomes in severe siderosis and early cardiac dysfunction.

*(b)*  *Iron chelation in Diabetes*

Across pancreatic, hepatic, intestinal, and renal contexts, convergent evidence demonstrates that iron overload and impaired antioxidant systems lead to ferroptosis, thereby undermining insulin secretion, insulin action, and organ integrity in diabetes (Table 2). Therapeutic strategies that (i) normalize systemic and cellular iron handling (e.g., chelators, PKCα inhibition), (ii) restore antioxidant/antiferroptotic defenses (e.g., GPX4/FSP1 pathways, GSH metabolism), and (iii) modulate upstream stress signaling (e.g., JAK2/STAT3, HIF-1α/NCOA4/FTH1) hold promise for slowing diabetes progression and mitigating its complications [27,31,32,33].

Preclinical models show that iron chelation improves glucose metabolism [40], wound healing [41], and nephropathy [32,40].

Several antidiabetic agents demonstrate anti-ferroptotic effects: metformin [41] reduces hepatic iron overload via AMPK–ferroportin signaling [42,43]; SGLT2 inhibitors [44,45] and GLP-1 receptor agonists attenuate ferroptosis in renal and cardiac tissues [46]; Dipeptidyl Peptidase-4 (DPP-4) inhibitors modulate ferroptosis via p53-dependent pathways [47]. Natural compounds (e.g., quercetin, curcumin) and experimental agents (GPX4/FSP1 activators) also show promise [48].

In animal models [27], the iron chelator DFX restored Janus Kinase 2/Signal Transducer and Activator of Transcription 3 (JAK2/STAT3) signaling, reduced lipid peroxidation, improved mitochondrial ultrastructure, and normalized glucose metabolism. These data support a model in which hepatic iron overload drives insulin resistance via ferroptosis mediated by JAK2/STAT3/SLC7A11 suppression, with DFX mitigating this cascade.

Pharmacologic PKC inhibition or PKCα knockout, across type 1 and type 2 diabetic mouse models and human duodenal biopsies [30], reduced Fpn abundance and normalized serum iron in diabetic and hemochromatosis settings, nominating PKCα as a tractable target for correcting iron dysregulation in metabolic disease.

In C57BL/6J mice and MIN6 β cells, Deng et al. [31] showed that iron overload impairs insulin secretion and disrupts glucose homeostasis through ferroptosis. DFX partially reversed these alterations—lowering lipid peroxidation and stress signaling—although tissue iron clearance was incomplete. These findings implicate iron-induced ferroptosis as a direct β-cell injury mechanism and highlight iron chelation and ferroptosis inhibition as therapeutic avenues.

In db/db mice and renal cells exposed to high glucose/high fat, Chen & Zhang [32] observed increased ferrous iron, lipid peroxidation, and mitochondrial damage consistent with ferroptosis, accompanied by dysregulated expression of NCOA4 and FTH1. Pharmacologic inhibition with YC-1 (a HIF-1 inhibitor) or treatment with metformin reduced HIF-1α/NCOA4 signaling, dampened lipid peroxidation, and protected renal tubular cells from ferroptosis. Collectively, these results position the NCOA4–FTH1 module as a central regulator of ferritinophagy-driven iron release that couples ferroptotic damage to fibrogenesis in DKD, suggesting therapeutic potential in targeting HIF-1α, NCOA4, or FTH1.

In streptozotocin-induced diabetic nephropathy (DN) in rats, DFO significantly improved renal function—lowering serum creatinine and blood urea nitrogen (BUN)—and ameliorated structural injury, including basement membrane thickening and podocyte foot process effacement [40]. DFO reduced renal iron burden (decreased FTH1 and hepcidin expression, restored ferroportin), preserved key slit diaphragm components (nephrin and podocin), and attenuated inflammatory and fibrotic signaling by downregulating IL-1β, NF-κB, Monocyte Chemoattractant Protein-1 (MCP-1), fibronectin, and collagen I. Histology corroborated reductions in inflammatory infiltrates and collagenous fibrosis. Even if systemic DFO use is constrained by toxicity and short half-life, localized delivery and optimized dosing may enhance clinical applicability.

In transfusion-dependent β-thalassemia major, severe iron overload (mean serum ferritin ≈ 3000 ng/mL) is tightly linked to impaired glucose homeostasis. In a cohort of 67 children, 16% exhibited dysglycemia at baseline (11.9% impaired fasting glucose, 10.4% impaired glucose tolerance, 1.4% diabetes), associated with higher ferritin and longer disease duration [49]. Low C-peptide levels suggested β-cell dysfunction as the primary driver, although homeostatic model assessment of insulin resistance (HOMA-IR) rose with increasing ferritin over time. Chelation influenced metabolic outcomes. DFP monotherapy significantly improved insulin resistance and reduced ferritin over six months, outperforming DFO or combination therapy—consistent with DFP’s intracellular iron chelation and potential mitigation of pancreatic iron toxicity. Complementing these observations, a phase IV study (NCT00800761, www.clinicaltrial.gov (accessed on 1 February 2026) conducted in Cagliari, Sardinia, compared combined oral DFP (75 mg/kg TID) plus subcutaneous DFO (40–50 mg/kg nightly, 5–7 days/week) versus DFO monotherapy in 28 adults with established cardiac iron overload. Over ~3.5 years, no cardiac events occurred in the combination arm in the high-ferritin subgroup, whereas four events, including three deaths, occurred in the DFO-only group (*p* = 0.0049). Combined chelation improved left ventricular ejection fraction and reduced serum ferritin without serious adverse events, suggesting enhanced cardiac iron clearance and functional benefit (NCT00800761).

In a diabetic mouse model and RAW264.7 macrophages, Cai et al. [41] showed dose-dependent acceleration of wound closure with DFO (nearly 88% closure at 14 days with 200 µM DFO vs. 62% in controls), improved epithelial regeneration and collagen deposition, and enhanced angiogenesis (CD31 expression and tubular structure formation). DFO shifted macrophage polarization from pro-inflammatory M1 (Inducible Nitric Oxide Synthase; iNOS+) toward reparative M2 (CD206+), mediated by increased heat shock protein (HSP70). Inhibition of HSP70 (knk437) reversed these effects, restoring NF-κB activity and favoring M1 polarization. Western blot analyses confirmed suppression of NF-κB signaling and upregulation of M2 markers, highlighting coordinated hypoxia and immune modulation in diabetic wounds.

Building on these findings, NCT03085771 is a phase II clinical trial evaluating topical DFO for chronic diabetic lower extremity ulcers, with endpoints including percent wound area reduction at 4 weeks, time to closure, angiogenesis markers, and safety (NCT03085771; clinicaltrials.gov). Intravenous DFO administered prior to controlled hypoxic exposure increased circulating endothelial progenitor cells and upregulated HIF-1α signaling relative to placebo, improving microvascular reactivity without serious adverse events [50] (NCT03085771). These data suggest that DFO can restore hypoxia signaling and enhance vascular adaptation in T1D, warranting larger and longer-term studies to establish clinical benefit.

In a human study, a single 450 mL phlebotomy increased insulin sensitivity by ~80% over four months in diabetics with high-ferritin [51]. The SAIGNEES trial (NCT01045525) also investigates phlebotomy for dysmetabolic iron overload but stresses the need for further outcome data. This trial evaluated whether sustained iron depletion through phlebotomy improves metabolic and hepatic outcomes in patients with dysmetabolic iron overload syndrome (DIOS). Over one year, maintaining serum ferritin below 50 µg/L led to a marked reduction in iron stores (mean ferritin 71 ± 48 µg/L vs. 733 ± 277 µg/L in controls). Despite effective iron removal, phlebotomy did not significantly improve fasting glucose, liver enzymes, or steatosis/fibrosis indices compared with lifestyle advice alone. HOMA-IR was paradoxically higher in the phlebotomy group, raising questions about metabolic benefits [52].

Despite encouraging experimental data, clinical translation remains limited, underscoring the need for mechanistic studies to refine molecular targets and optimize therapeutic protocols. Although clinical translation is still limited, targeting iron and inhibiting ferroptosis offers a novel, mechanism-based avenue to slow diabetes progression and mitigate organ complications.

**Table 2:** Summary of preclinical and clinical evidence evaluating iron chelation strategies in cardiometabolic diseases.

Therapeutic Context/Indication	Chelation/Strategy	Model/Population	Key Findings	Type of Study	Reference
Atherosclerotic vascular inflammation (LPS-induced)	DFO	Murine air-pouch; endothelial/immune assays	DFO inhibited p22phox, NADPH oxidase, ROS, NF-κB, VCAM-1/ICAM-1.	Animal model + in vitro	[37]
Anthracycline (DOX) cardiotoxicity	DFO (peri-infusion)	Pediatric cancer patients	Lower NT-proBNP; no toxicity; subtle echo improvements.	Randomized clinical trial	[38]
Cardiac iron overload in β-thalassemia	DFX vs. DFO	β-thalassemia major (*n* = 197)	DFX non-inferior to DFO for T2*; stable LVEF; similar safety.	Randomized clinical trial	[39]
Diabetic nephropathy (DN)	DFO	STZ-induced rat DN	DFO improved creatinine/BUN, inflammation, fibrosis; restored ferroportin.	Animal model	[40]
Anti-ferroptotic effect	Metformin	male Sprague-Dawley (SD) rats; Normal human hepatocytes cell line WRL68	↓iron overload via AMPK-ferroportin signaling	Animal model; in vitro	[42]
Ferroptosis inhibition	DFX	C57BL/6 J mice	↓iron-driven mitochondrial injury; ↑GPX4 and ↑SLC7A11; ↓PTGS2	Animal model	[27]
Alleviation of systemic iron overload	PKC inhibitor	Streptozotocin-induced Type 1 Diabetes mice; db/db mice and KKAy mice	↓ferroportin (Fpn) plasma-membrane trafficking; ↑Fpn internalization and degradation;	Animal model	[30]
Islet β cell ferroptosis	DFX	C57BL/6J mice; MIN6 β cells	Inhibition of iron-overload-induced ferroptosis via suppression of the ASK1/P-P38/CHOP pathway	Animal model + in vitro	[31]
Renal tubular cells protected from ferroptosis	YC-1; metformin	DKD mice and DKD cell model	↓HIF-1α and ↓NCOA4 expression, attenuating intracellular lipid peroxidation and ferroptosis	Animal model + in vitro	[32]
Diabetic wound healing	DFO	Diabetic mice + RAW macrophages	Accelerated closure; ↑angiogenesis; M1 → M2 shift; HSP70-dependent.	Animal model + in vitro	[41]
Iron chelation and glucose homeostasis	DFO, DFP,	β-Thalassemia Major pediatric patients	DFP improved glucose homeostasis—specifically insulin resistance and ferritin levels	Clinical trial	[49]
Microvascular reactivity in T1D	Topical/IV DFO	Human trial (Phase II)	↑EPCs, ↑HIF-1α, improved microvascular reactivity.	Clinical trial	[50]

## 4. Role of Copper and Cuproptosis in Cardiometabolic Diseases

Copper is an essential trace element required for mitochondrial respiration, antioxidant defense, and diverse enzymatic reactions, including the activity of superoxide dismutase (SOD), which limits oxidative injury in the vasculature [4,53]. Maintenance of copper homeostasis—coordinating intestinal absorption, systemic transport, tissue storage, and excretion—is therefore critical for vascular integrity. Perturbations in this balance exert a “double-edged sword” effect: copper deficiency compromises vascular elasticity and elevates arteriosclerosis risk, whereas copper excess amplifies oxidative and inflammatory cascades that accelerate atherosclerotic progression [4,53]. Recent work emphasizes copper metabolism as a determinant of endothelial function, extracellular matrix integrity, and lipid handling; notably, deficiency within the vessel wall coupled with excess in the circulation is associated with impaired endothelial repair, heightened oxidative stress, and accelerated plaque development [54].

*(a)*  *Mechanistic role of copper in CVDs*

Epidemiological and mechanistic data indicate that elevated serum copper correlates with increased cardiovascular mortality and plaque instability. Excess copper potentiates ROS generation, which drives lipid peroxidation, DNA damage, and formation of oxidized Low-Density Lipoprotein (ox-LDL). Copper surplus also activates NF-κB signaling, fostering pro-inflammatory cytokine expression and endothelial dysfunction [4]. Conversely, copper deficiency impairs antioxidant systems—most notably Cu/Zn-SOD and ceruloplasmin—reduces NO bioavailability, and perturbs lipid metabolism, culminating in hypercholesterolemia and endothelial dysfunction. Deficiency further engages inflammatory pathways and promotes adverse vascular remodeling [4,53]. Together, these observations underscore copper’s bidirectional influence on vascular health, necessitating physiologic range control to avert both oxidative toxicity and insufficiency-related vulnerability [4,53].

Copper dyshomeostasis contributes to atherosclerosis through convergent mechanisms. Oxidative stress arises from an imbalance between ROS production and antioxidant defenses, with copper excess catalyzing radical formation and lipid peroxidation. Inflammation is propagated by activation of NF-κB and Mitogen-Activated Protein Kinase (MAPK) pathways, increasing leukocyte recruitment and cytokine output. Endothelial dysfunction ensues via impaired NO signaling and upregulation of adhesion molecules, facilitating monocyte adhesion and transmigration. Disturbances in lipid metabolism include enhanced Low-Density Lipoprotein (LDL) oxidation and dysregulation of sterol regulatory element-binding protein (SREBP)-mediated fatty acid and cholesterol synthesis, reinforcing foam cell formation and plaque growth [4]. Integrating these axes, the restoration of copper homeostasis within atherosclerotic plaques has been proposed as a strategy to enable lesion regression—reframing therapy toward vascular rejuvenation and regeneration rather than mere disease attenuation [54].

**Scheme 4:**
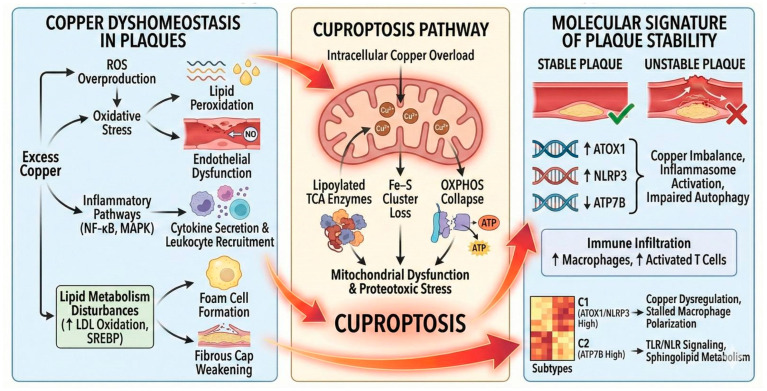
Copper imbalance and vascular pathology axes in atherosclerotic plaque progression. The schematic illustrates the interplay between copper dyshomeostasis, cuproptosis, and plaque stability. On the left, copper dyshomeostasis in plaques leads to excessive copper accumulation, promoting ROS overproduction, oxidative stress, and lipid peroxidation. These processes impair endothelial function (via nitric oxide reduction), activate inflammatory pathways (NF-κB, MAPK), and stimulate cytokine secretion and leukocyte recruitment. Copper imbalance also disrupts lipid metabolism (↑LDL oxidation, SREBP activation), contributing to foam cell formation and fibrous cap weakening. In the center, the cuproptosis pathway is triggered by intracellular copper overload, which binds lipoylated TCA cycle enzymes, causing Fe-S cluster loss, mitochondrial dysfunction, proteotoxic stress, and collapse of OXPHOS. On the right, the molecular signature of plaque stability contrasts stable plaques with unstable plaques characterized by copper imbalance, inflammasome activation, impaired autophagy, and immune infiltration (↑macrophages, ↑activated T cells). Key molecular markers include ATOX1, NLRP3, and ATP7B, with distinct immune subtypes (C1: ATOX1/NLRP3 high; C2: ATP7B high) linked to TLR/NLR signaling and sphingolipid metabolism. These mechanisms highlight copper dysregulation as a driver of vascular pathology and plaque destabilization.

Cuproptosis (Scheme 4) is a recently identified copper-dependent form of regulated cell death distinct from apoptosis, necroptosis, and ferroptosis. The ensuing proteotoxic stress drives cell death, directly linking copper overload to vascular damage and potentially to plaque progression [4]. As elaborated by He et al. [55], copper binding to mitochondrial lipoylated proteins derails the TCA cycle and cellular bioenergetics, introducing a mechanistic bridge between copper homeostasis, signaling pathway perturbation, and atherosclerotic pathogenesis.

Using Gene Expression Omnibus (GEO) datasets and machine learning, Muhetaer et al. [56] identified 27 differentially expressed cuproptosis-related genes (CRGs) in carotid plaques, with a five-gene diagnostic signature— Microtubule-Associated Protein 1 Light Chain 3 Alpha (LC3A), ATP7B, ATOX1, Copper Transporter 1 (CTR1), and NLRP3—distinguishing stable from unstable lesions. Unstable plaques exhibited upregulation of ATOX1 and NLRP3 and downregulation of ATP7B and LC3A, indicating copper dysregulation coupled with impaired autophagy and inflammasome activation. Functional enrichment linked these signatures to copper ion homeostasis, the mitochondrial matrix, and TCA cycle processes, aligning with cuproptosis hallmarks. Immune infiltration profiling revealed increased M0 macrophages and activated T cells in unstable plaques, consistent with a pro-inflammatory milieu. Consensus clustering further stratified unstable plaques into two subtypes: C1 (ATOX1/NLRP3-high), marked by copper imbalance and stalled macrophage polarization, and C2 (ATP7B-high), enriched for Toll-like and Nod-like receptor signaling, sphingolipid metabolism, and Peroxisome Proliferator-Activated Receptor (PPAR) pathways. Experimental validation confirmed ATOX1 and NLRP3 upregulation in unstable plaques and showed that ATOX1 inhibition (DC-AC50) rescued endothelial cells from copper-induced death, positioning ATOX1 as a functional regulator of cuproptosis. The study also points to ferroptosis crosstalk: ATP7B downregulation may impair ceruloplasmin activity, promoting iron accumulation and lipid peroxidation.

He et al. [55] analyzed carotid intimal hyperplasia (IH) using single-cell RNA sequencing, machine learning, and a rat balloon injury model, identifying Pyruvate Dehydrogenase Complex Component X (Pdhx) and Fdx1 as key CRGs. Both genes were downregulated in hyperplastic tissues (Neo) versus controls, with immunohistochemistry and external validation confirming these patterns. Diagnostic modeling achieved an Area Under the Curve (AUC) = 1.0, underscoring strong predictive value. Enrichment analyses implicated mitochondrial respiratory chain functions, NADH dehydrogenase assembly, oxidative phosphorylation, PI3K–Akt signaling, and Advanced Glycation End-product (AGE) pathways relevant to diabetic complications. Single-cell data revealed lower cuproptosis scores in Neo VSMCs, suggesting that suppressed cuproptosis facilitates VSMC proliferation; ultrastructural studies showed fewer mitochondrial alterations typical of cuproptosis in Neo tissues.

**Scheme 5:**
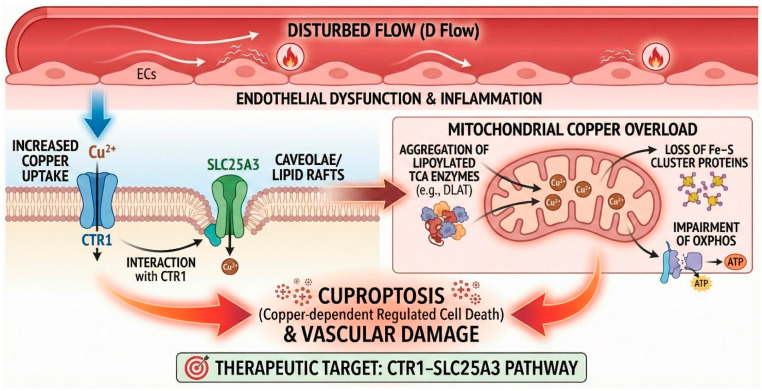
Disturbed flow and copper-driven endothelial injury in atherosclerosis. The schematic depicts the mechanistic link between hemodynamic disturbance, copper overload, and endothelial dysfunction leading to vascular damage. At the top, disturbed flow (D-flow) in endothelial cells (ECs) promotes inflammation and endothelial dysfunction. Increased copper uptake occurs via CTR1, which interacts with SLC25A3 in caveolae/lipid rafts, facilitating mitochondrial copper transport. Within mitochondria, copper overload induces aggregation of lipoylated TCA cycle enzymes (e.g., DLAT), loss of Fe-S cluster proteins, and impairment of oxidative phosphorylation (OXPHOS), ultimately triggering cuproptosis, a copper-dependent regulated cell death pathway. This process contributes to vascular damage and plaque instability. The figure highlights the CTR1–SLC25A3 axis as a potential therapeutic target for preventing copper-driven endothelial injury in atherosclerosis.

Atherosclerosis preferentially arises in arterial segments exposed to disturbed flow (D-flow), which provokes endothelial dysfunction, inflammation, and mitochondrial impairment (Scheme 5). Sudhahar et al. [57] delineate a copper-specific mechanism whereby D-flow increases endothelial copper uptake via the plasma membrane transporter CTR1 and promotes mitochondrial copper accumulation through the Mitochondrial Phosphate Carrier SLC25A3. Under D-flow, SLC25A3 relocates to caveolae/lipid rafts and interacts with CTR1, facilitating copper transfer to mitochondria. The resultant mitochondrial copper overload triggers aggregation of lipoylated proteins (e.g., DLAT), loss of Fe–S cluster proteins, and impairment of oxidative phosphorylation complexes—defining features of cuproptosis. Using synchrotron X-ray fluorescence microscopy for spatial mapping, Inductively Coupled Plasma Mass Spectrometry (ICP-MS) for compartmental quantification, Seahorse assays for respiration, and complementary genetic/pharmacologic interventions, the authors show that endothelial-specific CTR1 deletion in ApoE-deficient mice significantly reduces D-flow-induced atherosclerosis without altering lipid levels.

*(b)*  *Mechanistic role of copper in diabetes*

Growing evidence indicates that copper homeostasis is altered in T2DM, affecting both glycemic regulation and metabolic stability. The experimental human study [58] provides direct clinical evidence showing that 85.5% of T2DM subjects presented with copper deficiency, a condition associated with higher C-peptide levels, enhanced β-cell function (HOMA-B), and increased insulin resistance frequency when copper levels fell below 70 µg/dL. Despite this paradox, copper-deficient individuals exhibited a reduced likelihood of having fasting glucose > 130 mg/dL, suggesting a complex, compensatory metabolic interplay between copper availability, β-cell secretion, and glucose homeostasis.

Mechanistically, copper participates in redox biology as a cofactor of Cu/Zn SOD1, contributing to antioxidant defenses; however, both deficiency and overload may disrupt oxidative balance. Although the study found no significant relationship between copper levels and erythrocyte SOD activity, existing experimental work shows that copper imbalance can impair antioxidant capacity, increase ROS, and contribute to metabolic stress in diabetic tissues [58].

Several mechanistic studies highlight copper’s dual role in diabetes. Copper deficiency can impair glucose tolerance under certain dietary conditions. Experimental studies demonstrate that copper deficiency interacts with dietary carbohydrate composition to influence glucose homeostasis. In a classic experimental rat model, Fields et al. [59] showed that copper-deficient animals fed simple sugars (fructose or glucose) exhibit impaired glucose tolerance, characterized by elevated post-load glucose and reduced insulin responses, whereas starch-fed copper-deficient rats maintained normal glucose tolerance. This study identified a clear diet-dependent diabetogenic effect of copper deficiency, with fructose producing the most pronounced impairment.

Further mechanistic support comes from work in Cohen diabetic-sensitive rats [60], where a low-copper, high-sucrose diet precipitated hyperglycemia, impaired glucose-stimulated insulin secretion (GSIS), reduced cytochrome c oxidase activity, and pancreatic inflammatory changes. Copper supplementation not only prevented but also reversed these metabolic disturbances, highlighting copper’s essential role in maintaining mitochondrial function and β-cell stimulus–secretion coupling under high-carbohydrate conditions.

Complementary evidence from related dietary studies shows that fructose consumption exacerbates the severity of copper deficiency, leading to more profound metabolic and biochemical alterations than starch or glucose diets. This heightened susceptibility underscores that simple carbohydrates amplify the physiological consequences of copper depletion, making glucose tolerance particularly vulnerable in low-copper states [59].

Copper overload promotes pro-oxidant effects, mitochondrial dysfunction, and potential β-cell toxicity through cuproptosis. Evidence from dietary manipulation studies in susceptible diabetic rat models demonstrates that copper dysregulation impairs β-cell mitochondrial cytochrome c oxidase activity, reduces glucose-stimulated insulin secretion (GSIS), and promotes inflammatory infiltration of pancreatic islets—indicating a clear pathway by which copper-dependent mitochondrial dysfunction can translate into β-cell injury and metabolic deterioration [60]. Moreover, copper dysregulation contributes to insulin resistance through oxidative stress pathways, inflammatory signaling, and impaired insulin receptor function [58].

Overall, current experimental and mechanistic evidence indicates that copper dysregulation—whether deficiency or overload—disrupts metabolic homeostasis in diabetes, affecting β-cell function, oxidative balance, insulin sensitivity, and risk of complications (Table 3). However, the exact direction and impact of copper shifts appear strongly context-dependent, influenced by interactions with other minerals (e.g., zinc) [61], dietary factors [62], and the redox environment [63]. Further mechanistic studies and controlled clinical trials are needed to clarify copper-related therapeutic targets and to define optimal copper status thresholds for metabolic health in T2DM.

**Table 3:** The table summarizes major epidemiological, mechanistic, transcriptomic, and experimental evidence, as well as clinical findings, linking copper dyshomeostasis to vascular injury and atherosclerotic disease and to metabolic dysfunction in type 2 diabetes mellitus (T2DM).

Topic/Axis	Main Findings	Direction	Model	Mechanistic Notes	Reference
CRGs in plaque instability	27 CRGs identified; unstable plaques show ↑ATOX1/NLRP3, ↓ATP7B/LC3A.	Dyshomeostasis	Human plaque transcriptomics + ML	Inflammasome activation, impaired autophagy, immune infiltration.	[56]
Cuproptosis suppression in IH	Downregulation of Pdhx and Fdx1 → suppressed cuproptosis; VSMC proliferation.	Suppressed cuproptosis → progression	Single-cell + rat balloon injury	OXPHOS defects, PI3K–Akt, AGE pathways.	[53]
Disturbed flow induces Cu overload	D-flow increases endothelial uptake via CTR1 → mitochondrial Cu overload → cuproptosis.	Overload	In vitro flow + ApoE−/− mice	CTR1–SLC25A3 axis; rescues with CTR1 deletion.	[57]
Copper status altered in T2DM	85.5% of T2DM participants had Cu deficiency; altered C-peptide, HOMA-B, IR.	Deficiency	Human clinical study	Cu role via SOD1; no SOD correlation.	[58]
Diet × Cu deficiency	Fructose/glucose-fed Cu-deficient rats show impaired glucose tolerance.	Deficiency + simple sugars	Rat model	Diet-dependent diabetogenic effect.	[59]
Low-Cu high-sucrose diet	Hyperglycemia, reduced GSIS, low COX; reversed by Cu supplementation.	Deficiency under high-sucrose	Cohen diabetic-sensitive rat	COX + inflammation changes.	[60]
Fructose exacerbates Cu deficiency	More severe Cu deficiency phenotype with fructose.	Fructose worsens deficiency	Rat high-fructose diet	Greater metabolic disruption.	[59]

## 5. Copper Chelation in Cardiometabolic Diseases

The proposition that re-establishing copper homeostasis within atherosclerotic plaques could enable lesion regression reframes clinical objectives toward vascular rejuvenation [54]. Mechanism-based approaches include systemic or mitochondria-targeted copper chelation to quell cuproptosis and oxidative injury, alongside strategies that correct endothelial copper trafficking through CTR1/SLC25A3 modulation [57]. Given copper’s dual risks, therapeutic design should preserve physiological copper-dependent enzymatic functions (e.g., SOD, ceruloplasmin) while abrogating pathological mitochondrial copper accumulation and downstream proteotoxic stress [53].

Key priorities include defining the safe copper range for cardiovascular health; identifying cuproptosis-specific biomarkers for early detection and personalization; interrogating mitochondrial dynamics and mitophagy in copper-related vascular pathology; and developing combination therapies that concurrently target multiple cell death pathways, including cuproptosis, ferroptosis, and pyroptosis [4]. Recent conceptual advances emphasize the importance of copper homeostasis across signaling networks and cell death programs, pointing to new translational opportunities for cardiovascular disease management.

*(a)*  *Cooper chelation in CVDs*

Copper dyshomeostasis can promote atherogenesis through oxidative stress, endothelial dysfunction, and inflammatory signaling. Recent research has focused on therapeutic strategies that modulate copper homeostasis, including copper chelation (Table 4), regulation of copper chaperones, and copper-based nanomedicine [4].

In vivo mouse models by Sudhahar et al. [57] showed that copper chelation (e.g., tetrathiomolybdate) or mitochondria-targeted copper-depleting nanoparticles (mitoCDN) restore mitochondrial function, prevent DLAT aggregation, and decrease endothelial death and plaque formation. Inhibitors of alternative cell death pathways fail to block D-flow cytotoxicity, reinforcing the specificity of cuproptosis in this setting and highlighting the CTR1–SLC25A3 axis as a tractable target for copper-driven vascular injury.

Copper chelation represents the most extensively studied approach. Tetrathiomolybdate (TTM), a highly specific copper chelator originally developed for Wilson’s disease, has demonstrated significant anti-inflammatory and anti-atherogenic effects in preclinical models. Wei et al. [64] investigated TTM in apolipoprotein E-deficient mice, a well-established model of human atherogenesis. Dietary administration of TTM for ten weeks markedly reduced bioavailable copper, as evidenced by a 47% decrease in serum ceruloplasmin and an 80% reduction in the copper-to-molybdenum ratio in vascular tissues, without inducing hepatotoxicity or severe anemia. Copper depletion was associated with substantial anti-inflammatory effects, including reductions in soluble adhesion molecules Vascular Cell Adhesion Molecule (VCAM-1) and Intercellular Adhesion Molecule-1 (ICAM-1), downregulation of pro-inflammatory genes such as MCP-1, TNF-α, and IL-6, and attenuation of macrophage infiltration. Mechanistically, these changes were linked to inhibition of NF-κB-mediated transcriptional activation, thereby limiting monocyte recruitment and vascular inflammation. Morphometric analysis revealed a 25% reduction in overall lesion area and a 45% reduction in the descending aorta, suggesting that copper chelation primarily inhibits lesion initiation rather than progression. Lipid alterations were modest, indicating that TTM’s anti-atherogenic effect is not mediated by lipid lowering.

Additional studies have confirmed the anti-inflammatory properties of TTM in different contexts. Wei et al. [65] demonstrated that TTM suppresses LPS-induced systemic inflammation in C57BL/6N mice by reducing serum levels of MCP-1, IL-1α, and TNFα, alongside downregulation of adhesion molecules in cardiovascular tissues. These effects were mediated through inhibition of NF-κB and AP-1 activation, independent of antioxidant enzyme modulation. In vitro experiments further showed that copper exposure activates NF-κB and Activator Protein-1 (AP-1) signaling in human aortic endothelial cells, increasing VCAM-1, ICAM-1, and MCP-1 expression, whereas TTM completely abolished these responses. Collectively, these findings establish copper as a pro-inflammatory signal and position TTM as a potent inhibitor of endothelial activation.

Copper chelation also influences vascular remodeling. Mandinov et al. [66] reported that TTM attenuates neointimal thickening in rat carotid balloon injury models by inhibiting copper-dependent secretion of IL-1 and Fibroblast Growth Factor 1 (FGF1), reducing monocyte recruitment, and accelerating reendothelialization. These observations suggest that copper chelation may serve as an anti-restenotic strategy with potential applications in angiogenesis-driven diseases. Although Ethylenediaminetetraacetic Acid (EDTA) chelation has been evaluated in clinical trials for post-myocardial infarction patients, results remain inconclusive, underscoring the need for more targeted copper-specific approaches [4].

Beyond chelation, modulation of copper chaperones represents an emerging therapeutic avenue. Proteins such as ATOX1, which interact with TNF Receptor-Associated Factor 4 (TRAF4), influence endothelial activation and vascular smooth muscle cell migration, processes central to atherogenesis. Targeting these interactions may mitigate vascular inflammation and remodeling, although this strategy remains in early experimental stages [4].

Innovative approaches involving copper ionophores and nanomedicine are also under investigation. Agents such as elesclomol [67] and copper-based nanoparticles [68] aim to exploit copper’s role in regulated cell death pathways, particularly cuproptosis. Cuproptosis is tightly linked to mitochondrial dysfunction, which plays a pivotal role in plaque formation and instability.

Interestingly, recent evidence challenges the traditional paradigm of copper depletion as the sole therapeutic goal. Zuo et al. [54] propose restoring copper specifically to Cu-deficient vascular tissue as a means to reverse atherosclerosis. Using an ultrasound-assisted copper–albumin microbubble delivery system (Cu-MB-US), targeted copper repletion in rabbit models significantly reduced plaque area, restored vascular copper levels, improved endothelial survival, and lowered lipid content without destabilizing plaques. These findings highlight the therapeutic potential of precision copper delivery for vascular regeneration, contrasting with conventional dietary supplementation, which often elevates serum copper without replenishing vascular stores and may exacerbate oxidative stress.

**Table 4:** The table summarizes current experimental and emerging therapeutic strategies aimed at modifying copper homeostasis to counteract atherosclerosis and vascular inflammation. These approaches are grounded in mechanistic evidence showing that copper dyshomeostasis promotes oxidative stress, endothelial dysfunction, inflammatory activation, and cuproptosis-mediated cell death, all of which contribute to plaque formation and vascular injury.

Intervention/Strategy	Agent/Target	Disease Context and Model	Key Outcomes	Mechanistic Readouts	Stage	Ref.
Chelation: flow-induced cuproptosis	TTM, mitoCDN	Disturbed-flow mouse models	Restores mitochondrial function; prevents DLAT aggregation; ↓endothelial death and plaques.	CTR1–SLC25A3 axis; suppression of lipoylated protein aggregation.	Preclinical	[57]
Chelation: anti-atherogenic efficacy	TTM	ApoE-/- mice	↓Cu availability; ↓VCAM-1/ICAM-1; ↓MCP-1/TNF-α/IL-6; ↓macrophage infiltration; ↓lesion area.	NF-κB inhibition; anti-inflammatory.	Preclinical	[64]
Chelation: systemic inflammation and endothelial activation	TTM	C57BL/6N mice + HAECs	↓MCP-1, IL-1α, TNF-α; ↓adhesion molecules; TTM abolishes Cu-induced NF-κB/AP-1.	NF-κB/AP-1 suppression.	Preclinical	[69]
Chelation: restenosis	TTM	Rat balloon injury model	↓Neointima; ↓IL-1/FGF1; ↓monocyte recruitment; faster re-endothelialization.	IL-1/FGF1 inhibition.	Preclinical	[66]
Non-specific chelation	EDTA	Post-MI patients (clinical trials)	Mixed/inconclusive effects.	Non-specific metal chelation.	Clinical	[4]
Precision copper repletion	Cu-MB-US	Rabbit atherosclerosis	↓Plaque area; restored vascular Cu; ↑endothelial survival; ↓lipid content.	Targeted Cu restoration; re-endothelialization.	Preclinical	[54]

*(b)*  *Copper chelation in Diabetes*

Chang et al. [70] reviewed experimental studies on copper chelation in diabetes, emphasizing its role in mitigating oxidative stress and preventing complications such as neuropathy, nephropathy, and retinopathy. Copper dysregulation promotes ROS generation and activates inflammatory pathways, contributing to tissue damage. Chelators like trientine and TTM restore copper balance, reduce oxidative burden, and improve organ function. Emerging evidence also implies cuproptosis in diabetic complications, suggesting that copper-targeted therapies may modulate cell survival pathways. These mechanistic insights underscore the therapeutic potential of copper chelation beyond glycemic control, positioning it as a strategy for systemic protection in diabetes.

Tanaka et al. [71] investigated the role of copper chelation in type 2 diabetes using C57BL/KsJ-db/db mice, a well-established model of diabetic pathology. These mice exhibit hyperglycemia, insulin resistance, and dyslipidemia, conditions often associated with elevated oxidative stress. The authors hypothesized that copper ions, as redox-active metals, exacerbate oxidative damage and metabolic dysfunction in diabetes. TTM, a potent copper chelator, was administered orally to diabetic mice for several weeks. Treatment significantly reduced serum copper levels and ROS, indicating effective chelation and oxidative stress mitigation. Importantly, TTM improved glucose tolerance and insulin sensitivity, as evidenced by lower fasting glucose levels and enhanced insulin signaling. Lipid profiles also improved, with reductions in triglycerides and partial normalization of cholesterol levels. Histological analysis revealed decreased inflammatory infiltration in pancreatic tissue and improved β-cell morphology. These findings suggest that copper contributes to the pathogenesis of type 2 diabetes through oxidative and inflammatory mechanisms and that chelation therapy can restore metabolic homeostasis without overt toxicity.

Cooper et al. [72,73] explored the therapeutic potential of trientine, a potent copper chelator [74] clinically approved for Wilson’s disease, in diabetic cardiomyopathy. In streptozotocin (STZ)-induced diabetic rats, oral trientine administration increased urinary copper excretion and led to remarkable improvements in cardiac structure and function. Treated animals exhibited reduced left ventricular fibrosis, improved cardiomyocyte ultrastructure, and decreased β1 integrin expression, a marker of pathological remodeling. These changes occurred independently of glycemic control, indicating a direct effect of copper chelation on cardiac tissue. Extending these findings to humans, the authors conducted a pilot study in patients with type 2 diabetes and left ventricular hypertrophy. After six months of trientine therapy, participants showed significant reductions in left ventricular mass, suggesting partial reversal of diabetic cardiomyopathy. These results highlight copper chelation as a novel strategy for managing cardiovascular complications in diabetes, with benefits beyond glucose regulation [72,73].

Building on preclinical evidence, a double-blind, placebo-controlled randomized trial evaluated trientine in patients with type 2 diabetes and left ventricular hypertrophy. Participants received oral trientine (600 mg twice daily) or placebo for 12 months. The primary endpoint was change in left ventricular mass indexed to body surface area, assessed by cardiac imaging. Trientine treatment resulted in a progressive reduction in ventricular mass—approximately 5 g/m^2^ at six months and 10.6 g/m^2^ at 12 months—while no significant change occurred in the placebo group. Urinary copper excretion correlated strongly with ventricular mass reduction, confirming the mechanistic link to copper chelation. Importantly, these cardiac benefits were independent of changes in blood glucose or blood pressure, suggesting that copper chelation exerts direct effects on myocardial remodeling. The trial provides compelling evidence for the clinical utility of trientine in reversing structural cardiac changes in diabetic patients [72].

Experimental and clinical evidence strongly supports copper chelation as a promising intervention in type 2 diabetes (Table 5). Animal studies demonstrate that chelators such as TTM and trientine improve glucose metabolism, reduce oxidative stress, and protect pancreatic and cardiac tissues. Human trials confirm that trientine reverses left ventricular hypertrophy, a major cardiovascular complication of diabetes, through mechanisms independent of glycemic control. Mechanistic reviews highlight copper’s role in oxidative stress, inflammation, and cell death pathways, providing a rationale for targeting copper homeostasis. While these findings are encouraging, further research is needed to define optimal dosing, long-term safety, and the broader impact on diabetic complications. Copper chelation therapy represents an innovative approach that addresses the underlying pathophysiology of diabetes rather than focusing solely on glucose regulation.

## 6. Cross-Talk of Copper and Iron in Cardiometabolic Diseases

The interplay between copper and iron represents a critical axis in the pathophysiology of cardiometabolic disorders. Both metals are essential cofactors for numerous enzymatic processes, yet their dysregulation transforms them into potent drivers of oxidative stress, mitochondrial dysfunction, and inflammatory signaling. This dual-metal imbalance underpins key mechanisms such as ferroptosis and cuproptosis—two regulated cell death programs increasingly recognized as central to cardiometaboolic disease progression.

Oxidative stress is no longer considered a mere imbalance between oxidants and antioxidants but rather a disruption of a complex redox network that integrates metabolism, immunity, and metal homeostasis. Within this framework, ferroptosis and cuproptosis emerge as two regulated, metal-dependent forms of cell death that exemplify how dysregulated iron and copper fluxes convert adaptive redox signaling into cytotoxicity.

Cuproptosis/ferroptosis cross-talk manifests in diverse clinical contexts. In atherosclerosis, iron-driven lipid oxidation destabilizes plaques [23], while copper dysregulation perturbs endothelial function and immune signaling [75]. Hypertension involves AngII-mediated oxidative stress [76] and GPX4 suppression [77], intersecting with copper efflux pathways that modulate vascular remodeling. Myocardial infarction illustrates copper’s dual role: it promotes angiogenesis via HIF-1α and VEGF but exacerbates oxidative injury and cuproptosis when compartmental control fails [78,79]. Heart failure and DiaCM further highlight mitochondrial vulnerability, where iron overload and copper mismanagement converge to impair energy metabolism and accelerate cardiomyocyte loss.

Despite these convergences, ferroptosis and cuproptosis diverge in execution chemistry and spatial bias. Ferroptosis depends on polyunsaturated phospholipid peroxidation and GPX4 failure, whereas cuproptosis requires copper engagement with the lipoylated proteome and subsequent proteotoxic aggregation. Spatially, ferroptosis is membrane lipid-centric, affecting plasma and mitochondrial membranes, while cuproptosis is confined to the mitochondrial matrix, targeting the TCA lipoyl.

In diabetes, hyperglycemia-driven ROS elevation and impaired antioxidant defenses sensitize β-cells to ferroptosis and cuproptosis [78,80]. Cardiovascular pathology similarly reflects iron-driven Lipid Peroxidation (LPO) and copper-mediated mitochondrial failure, suggesting shared metabolic hubs such as the TCA cycle and GSH-dependent redox buffering.

Across the cardiovascular continuum—from hypertension and atherosclerosis to MI, HF, and diabetes-related cardiac remodeling —iron and copper dysregulation orchestrate overlapping pathogenic cascades. Iron-driven lipid peroxidation injures endothelial cells, vascular smooth muscle cells, and cardiomyocytes, promoting plaque instability, maladaptive remodeling, and HF, while copper imbalance impairs mitochondrial enzymes, boosts ROS production, and alters angiogenesis and fibrosis [2,4]. In atherosclerosis, ferroptosis accelerates lipid oxidation and plaque vulnerability, while cuproptosis-related genes such as FDX1, SLC31A1, and GLS appear in plaque datasets, suggesting diagnostic potential [2]. Hypertension exhibits evidence of ferroptotic remodeling through AngII-mediated IL-6/STAT3 signaling and GPX4 suppression, alongside cuproptosis links involving SIRT7 and ATP7A [2]. In HF, mitochondrial centrality dominates: iron overload drives mitochondrial ferroptosis, while FDX1 emerges as a cuproptosis target. Chelators may restore mitochondrial programs and improve cardiac energetics [2]. MI illustrates copper’s ambivalence: it promotes angiogenesis via HIF-1/VEGF signaling but also triggers oxidative stress and cuproptosis when compartmental control fails. Copper influences fibrosis through lysyl oxidase and Matrix Metalloproteinase (MMP) activity, modulates cytochrome c oxidase and SOD-dependent energetics, and shapes oxidative signaling. Clinical studies reveal heterogeneous copper patterns, and therapeutic strategies range from chelation to ionophore-mediated delivery [4].

Therapeutic principles converge on rebalancing metals, protecting membranes, and preserving mitochondrial integrity. Ferroptosis blockade employs radical-trapping antioxidants such as Fer-1 and Lip-1-1, iron chelators like DFO, GPX4 and FSP1 support, and Nrf2 activation. Cuproptosis modulation relies on copper chelators (trientine, tetrathiomolybdate) to curb overload, ionophores (elesclomol) for targeted delivery, and respiratory inhibitors to attenuate execution. Targeting the FDX1-lipoylation axis offers mechanistic specificity [4,5]. In MI, copper supplementation rescues angiogenesis but risks oxidative injury; benefit–risk profiles hinge on timing, dose, and compartmentalization. Strategies include chelators for overload, ionophores for deficiency, and nanoplatforms such as Cu-ceria particles to enhance antioxidant defenses and perfusion [4].

Pulmonary hypertension (PH) is a severe disorder marked by progressive vascular remodeling, oxidative stress, and high mortality. Recent research [81] has uncovered the involvement of both ferroptosis and cuproptosis in its pathogenesis. Both pathways disrupt cellular homeostasis and energy metabolism, contributing to pulmonary vascular remodeling. The interplay between ferroptosis and cuproptosis appears to be significant. Copper can promote GPX4 degradation via autophagy, while iron–sulfur clusters act as regulatory hubs linking these processes. Hypoxia, inflammation, and metabolic reprogramming further amplify this crosstalk, suggesting a shared pathogenic network. Therapeutically, ferroptosis inhibitors such as Fer-1 and Coenzyme Q10, as well as copper chelators like tetrathiomolybdate and trientine, have shown promise in preclinical models [80,82]. Emerging strategies aim to co-target both pathways using nanocarriers or combined small molecules, though clinical evidence remains limited. Understanding these mechanisms opens new perspectives for PH treatment [40].

**Scheme 6:**
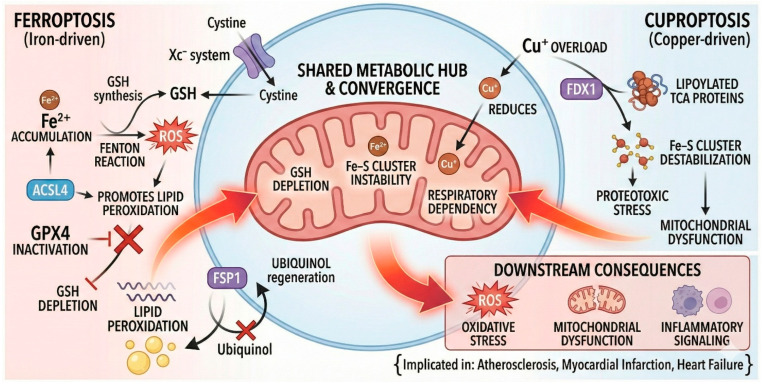
Interconnected Ferroptosis and Cuproptosis Pathways. The schematic illustrates the convergence of iron-driven ferroptosis and copper-driven cuproptosis at the mitochondrial metabolic hub. Ferroptosis is characterized by Fe^2+^ accumulation, Fenton reaction-mediated reactive oxygen species (ROS) generation, glutathione (GSH) depletion, and GPX4 inactivation, leading to lipid peroxidation. Cuproptosis involves Cu^+^ overload, reduction by FDX1, and binding to lipoylated TCA cycle proteins, causing Fe-S cluster destabilization and proteotoxic stress. Both pathways share mitochondrial vulnerability, including Fe-S cluster instability and respiratory dependency. Downstream consequences include oxidative stress, mitochondrial dysfunction, and inflammatory signaling, which are implicated in cardiovascular pathologies such as atherosclerosis, myocardial infarction, and heart failure. Key regulators highlighted include ACSL4, FSP1, and FDX1.

All papers converge on the idea that metal-dependent regulated cell death (RCD)—primarily ferroptosis (iron-driven lipid peroxidation) and cuproptosis (copper-driven, lipoylation-dependent mitochondrial proteotoxicity)—is not an epiphenomenon but a mechanistic driver across cardiovascular diseases (CVD) and beyond (Scheme 6).

Therapeutically, dual-action chelators capable of binding both copper and iron offer a rational approach to mitigate these intertwined pathways. Traditional agents such as DFO, primarily an iron chelator, exhibit secondary copper-binding properties and could be repurposed for ischemia–reperfusion injury. Rationally designed ligands and polymer-based chelators promise improved selectivity and pharmacokinetics, reducing systemic toxicity while targeting pathological metal pools. By lowering both copper- and iron-driven radical formation, these agents may attenuate ferroptotic and cuproptotic cell death, preserve mitochondrial integrity, and improve vascular and cardiac outcomes.

However, challenges remain. Achieving metal selectivity without depleting essential cofactors, ensuring compartment-specific delivery, and validating efficacy through clinical trials are critical steps. Integration of redox lipidomics and metallomics with machine-learning models may enable patient stratification and guide personalized chelation strategies. Ultimately, the copper–iron axis represents not only a mechanistic bridge between metabolic stress and regulated cell death but also a therapeutic frontier for precision redox cardiology.

## 7. Biomarker Translation Gaps: Assay Limitations, Compartment Specificity, and the Absence of Validated Cuproptosis Markers

Although ferroptosis and cuproptosis have rapidly gained prominence as mechanistic drivers of cardiometabolic disease, the translation of their biomarkers into clinically actionable tools remains challenged by several under-underrecognized limitations. First, the analytical foundation itself is fragmented. Most biomarkers associated with ferroptosis—such as GPX4 abundance, ACSL4 expression, PTGS2 induction, or panels of oxidized phospholipids—depend on research-grade platforms including redox lipidomics, high-resolution mass spectrometry, or advanced proteomics. These technologies are not harmonized across laboratories, lack standardized internal controls, and are rarely adaptable to routine diagnostic workflows. Consequently, even widely referenced systemic indices such as serum copper, ceruloplasmin, ferritin, or transferrin saturation do not reliably reflect the dynamic and compartmentalized redox states that determine whether ferroptosis or cuproptosis is engaged in tissue. Their variability across cohorts, combined with susceptibility to dietary, inflammatory, and metabolic confounders, further undermines their translational utility—an issue highlighted in recent analyses across cardiovascular and metabolic disease populations [83,84].

A second and equally important challenge arises from the profound compartment specificity of both pathways, which is not captured by existing clinical assays. Ferroptosis and cuproptosis are inherently spatially constrained processes: the former reflects iron-driven lipid peroxidation occurring at the plasma membrane and within mitochondria, whereas the latter is entirely dependent on mitochondrial copper binding to the lipoylated tricarboxylic acid (TCA) proteome. However, clinical biomarkers remain primarily systemic. They cannot distinguish between cytosolic versus mitochondrial metal overload, nor between endothelial, smooth muscle, immune-cell, or cardiomyocyte vulnerability. Elegant mechanistic studies show that endothelial mitochondrial copper accumulation can occur in disturbed-flow states even when serum copper remains unchanged, underscoring the disconnect between circulating markers and the intracellular metal pools that actually trigger cuproptosis. This spatial mismatch significantly limits the clinical interpretability of systemic metal measurements and underscores the need for organelle-resolved diagnostics [83,85].

A third barrier is the absence of validated cuproptosis-specific biomarkers, which remains a substantial translational bottleneck. While ferroptosis biomarkers have undergone partial validation in cardiac, vascular, and metabolic tissues, cuproptosis—which hinges on copper-dependent aggregation of lipoylated mitochondrial proteins and Fe–S cluster destabilization—lacks any accepted biochemical surrogate. Proposed markers such as FDX1 activity, DLAT or DLST lipoylation status, ATP7A/B expression, ATOX1 upregulation, or CTR1-dependent copper flux have been identified in transcriptomic and proteomic studies of atherosclerosis, pulmonary hypertension, and myocardial infarction, but none have been standardized, quantified in large human cohorts, or shown to predict clinical outcomes. Moreover, many of these markers respond to broader mitochondrial stress and are not specific to cuproptosis, further complicating their interpretation. Until assays can directly measure lipoylated protein aggregation or mitochondrial copper overload in accessible clinical samples, the mechanistic richness of cuproptosis will remain difficult to harness diagnostically [86].

Finally, the literature is marked by methodological variability across preclinical models, tissue types, and analytical platforms, which compounds the above limitations. Differences in animal strain, diet, disease stage, metabolic context, redox environment, and experimental assay selection generate heterogeneity that limits reproducibility and obscures consensus on biomarker thresholds or clinical significance. This variability has been repeatedly highlighted in ferroptosis-related cardiovascular studies, and it is even more pronounced in cuproptosis research, which remains at an earlier developmental stage with fewer validated endpoints. The absence of prospective, biomarker-anchored clinical trials further widens this translational gap [87].

Together, these limitations underscore that while ferroptosis and cuproptosis hold great promise as diagnostic and therapeutic targets, their biomarker landscape remains insufficiently structured. Future progress will require coordinated standardization of assays, development of compartment-specific measurement tools—particularly for mitochondrial metal pools—and rigorous clinical validation of candidate gene or protein signatures. Only then can ferroptosis and cuproptosis biomarkers be reliably integrated into precision redox cardiology.

## 8. Conclusions

Cardiometabolic diseases are increasingly understood as disorders of metal-dependent redox imbalance, where iron-driven ferroptosis and copper-driven cuproptosis act as central, mitochondria-focused mechanisms of vascular and cardiac injury. These pathways share upstream vulnerabilities—glutathione depletion, Fe–S cluster instability, and TCA cycle dependence—while diverging in execution chemistry and spatial localization. Their convergence amplifies oxidative stress, inflammation, and metabolic failure across hypertension, atherosclerosis, myocardial infarction, heart failure, and diabetic cardiomyopathy.

Mechanistic markers such as GPX4/SLC7A11 loss, ACSL4 expression, FDX1 signaling, and DLAT lipoylation, combined with redox lipidomics and metallomics, offer a foundation for integrated “redox phenotyping.” Multi-omic machine-learning signatures (e.g., STK17B, NLRP1, PELI1) show strong predictive value for CAD, but clinical translation requires harmonized panels and prospective validation.

Strategies converge on restoring metal balance and mitochondrial integrity. Ferroptosis inhibitors, iron chelators, and antioxidants complement copper chelators, ionophores, and mitochondria-targeted delivery systems. Dual-action approaches—co-targeting iron and copper—may outperform single-pathway interventions, particularly in conditions dominated by mitochondrial dysfunction. Precision timing is critical: copper supplementation can aid angiogenesis post-MI, while chelation prevents cuproptotic injury.

In summary, ferroptosis and cuproptosis represent actionable targets for precision redox cardiology. Moving from mechanistic insight to clinical impact will require multi-omic diagnostics, dual-pathway therapeutics, and rigorously designed trials to reduce residual cardiovascular risk and improve outcomes.

## 9. Future Directions

Future research should focus on translating mechanistic insights into clinically actionable strategies. First, standardizing redox phenotyping is essential: integrating lipidomics, metallomics, and gene signatures into validated panels will enable precise risk stratification across conditions such as CAD, MI, HF, and diabetes-related cardiac remodeling. These tools should be linked to clinical outcomes and incorporated into prospective studies.

Second, compartment-specific therapeutics must be prioritized. Mitochondria-targeted copper depletion, endothelial-focused iron modulation, and lipophilicity-tuned radical-trapping antioxidants represent promising approaches to minimize systemic toxicity while maximizing efficacy. Nanotechnology-based carriers and controlled-release systems will play a key role in achieving this precision.

Third, dual-pathway interventions—co-targeting ferroptosis and cuproptosis—should be tested in biomarker-guided trials. Combining iron chelators with copper chelators or ionophores, alongside mitochondrial support, may offer synergistic benefits in advanced cardiometabolic disease. These trials should incorporate adaptive designs and AI-driven analytics to optimize dosing and predict responders.

Fourth, bioinformatics-driven drug repositioning and machine-learning models should be expanded to identify novel compounds and match them to patient-specific immune–redox profiles. Genes such as STK17B, NLRP1, and PELI1, already linked to CAD, warrant functional validation as therapeutic targets.

Finally, the timing and context of intervention must be clarified. For example, copper supplementation may enhance angiogenesis during early MI recovery, whereas chelation is critical to prevent cuproptotic injury in later stages. Defining these therapeutic windows will be key to safe and effective implementation.

In summary, the next decade should see a shift toward precision redox cardiology, combining multi-omics diagnostics, targeted therapeutics, and AI-guided personalization to reduce residual cardiovascular risk and improve patient outcomes.

## 10. Limitations

Despite growing mechanistic evidence, several limitations constrain the translation of ferroptosis–cuproptosis research into clinical practice. First, most current data derive from in vitro experiments and animal models, which do not fully replicate the complexity of human cardiometabolic disease. The interplay between iron and copper homeostasis, mitochondrial dynamics, and immune signaling in patients remains incompletely understood.

Second, biomarker specificity and standardization are major challenges. While markers such as GPX4, FDX1, DLAT lipoylation, and oxidized lipid panels show promise, they lack validated clinical thresholds and are not routinely available in diagnostic workflows. Similarly, serum copper and iron indices exhibit high inter-individual variability and are influenced by diet, inflammation, and comorbidities, limiting their predictive accuracy.

Third, therapeutic strategies face safety and feasibility concerns. Chelators and ionophores can disturb essential metal balance, cause systemic toxicity, and require precise compartmental targeting to avoid adverse effects. Nanocarrier-based delivery systems and mitochondria-specific interventions remain experimental, with limited long-term safety data.

Fourth, clinical trial evidence is scarce. Most proposed interventions—such as dual-action chelation or ferroptosis inhibitors—lack large-scale, randomized studies. Existing trials often focus on surrogate endpoints rather than hard cardiovascular outcomes, making it difficult to assess real-world benefit.

Finally, integration into clinical care pathways poses logistical and economic hurdles. Advanced diagnostics like redox lipidomics and metallomics demand specialized infrastructure and expertise, which may not be accessible in resource-limited settings. Without cost-effective and scalable solutions, widespread adoption will remain challenging.

In summary, while targeting ferroptosis and cuproptosis offers exciting therapeutic potential, progress depends on bridging these gaps through robust clinical validation, biomarker standardization, and development of safe, affordable interventions.

## Figures and Tables

**Table 5:** This table provides a synthesized overview of therapeutic strategies that target copper homeostasis across vascular disease, atherosclerosis, and cardiometabolic pathology. It integrates conceptual frameworks, mechanistic discoveries, preclinical interventions, and clinical trial evidence, illustrating how copper imbalance—both overload and deficiency—becomes a tractable therapeutic axis.

Therapeutic Context/Indication	Chelation/Strategy	Model/Population	Key Findings	Type of Study	Underlined Study/Trial
Copper-driven vascular injury	TTM or mitoCDN	ApoE-KO mice; EC-Ctr1 KO	Chelation or mitoCDN restores mitochondrial function, prevents DLAT aggregation, reduces endothelial death and plaque formation.	Animal model	Sudhahar et al. [32]
Correcting endothelial copper trafficking	CTR1/SLC25A3 modulation	Disturbed-flow EC models	CTR1–SLC25A3 axis regulates mitochondrial copper overload and cuproptosis.	Mechanistic study	Sudhahar et al. [32]
Diabetic cardiomyopathy	Trientine	Diabetic rats	Reduced Left Ventricle (LV) fibrosis; improved myocardial structure; increased Cu excretion; benefits independent of glycemia.	Animal model	Cooper et al. [36]
Type 2 diabetes with LV hypertrophy	Trientine	Human RCT	Regression of LV mass; changes linked to copper removal rather than glucose control.	Randomized clinical trial	Cooper et al. [38]

## Data Availability

No new data were created or analyzed in this study. Data sharing is not applicable to this article.

## Abbreviations

AC50—Half-maximal activity concentration; ACSL4—Acyl-CoA Synthetase Long-Chain Family Member 4; AGE—Advanced Glycation End-product; AI—Artificial Intelligence; AMP—Adenosine Monophosphate; AMPK—AMP-activated Protein Kinase; AngII—Angiotensin II; AP-1—Activator Protein-1 transcription factor; ASK1—Apoptosis Signal-Regulating Kinase 1; ATOX1—Antioxidant 1 Copper Chaperone; ATP7A/ATP7B—Copper-transporting ATPases 7A and 7B; AUC—Area Under the Curve; BUN—Blood Urea Nitrogen; C1/C2—Molecular clusters/subtypes of unstable plaques; C57BL/6J—C57BL/6J mouse strain; CAD—Coronary Artery Disease CD206—Macrophage Mannose Receptor (M2 phenotype marker); CD31—Platelet/Endothelial Cell Adhesion Molecule-1 (PECAM-1); CFP—Cuproptosis/Ferroptosis/Pyroptosis composite signature; CHOP—C/EBP Homologous Protein; CI—Confidence Interval; CMDs—Cardiometabolic diseases; CORDELIA—Clinical trial acronym (DFX vs. DFO in thalassemia); CRGs—cuproptosis-related genes ; CTD—C-terminal DomainCTDSP2—CTD Small Phosphatase 2; CTR1—Copper Transporter 1; CVD—Cardiovascular Disease; DBT—Dihydrolipoamide Branched-Chain Transacylase E2; DFO—Deferoxamine; DFP—Deferiprone; DFX—Deferasirox, DHODH—Dihydroorotate Dehydrogenase; DHRS7—Dehydrogenase/Reductase 7; DiaCM—Diabetic Cardiomyopathy; DLAT—Dihydrolipoamide Acetyltransferase; DLST—Dihydrolipoamide Succinyltransferase; DMT1—Divalent Metal Transporter 1; DN—Diabetic Nephropathy; DOX—Doxorubicin; DPP-4—Dipeptidyl Peptidase-4; EC—Endothelial Cell; EDTA—Ethylenediaminetetraacetic Acid; eNOS—Endothelial Nitric Oxide Synthase; ER—Endoplasmic Reticulum; FDX1—Ferredoxin 1; Fe–S—Iron–Sulfur cluster; Fer-1—Ferrostatin-1; FGF1—Fibroblast Growth Factor 1; Fpn—Ferroportin (SLC40A1); FSP1—Ferroptosis Suppressor Protein 1; FTH1—Ferritin Heavy Chain; GCSH—Glycine Cleavage System H-Protein; GLP-1—Glucagon-Like Peptide-1; GLS—Glutaminase; GPX4—Glutathione Peroxidase 4; GSH—Glutathione; GLUT4—Glucose Transporter Type 4; HbA1c—Glycated Hemoglobin A1c; HF—Heart Failure; HIF-1α—Hypoxia-Inducible Factor-1 alpha; HOMA-IR—Homeostatic Model Assessment of Insulin Resistance; HSP70—Heat Shock Protein 70; ICAM-1—Intercellular Adhesion Molecule-1; ICP-MS—Inductively Coupled Plasma Mass Spectrometry; IH—Intimal Hyperplasia; IL-6—Interleukin-6; IR—Insulin Resistance; iNOS—Inducible Nitric Oxide Synthase, JAK2—Janus Kinase 2; JUNB—JunB Proto-Oncogene; LC3A—Microtubule-Associated Protein 1 Light Chain 3 Alpha; LDL—Low-Density Lipoprotein; Lip-1—Liproxstatin-1; LPS—Lipopolysaccharide; LPO—Lipid Peroxidation ; LV—Left Ventricle; LVEF—left ventricular ejection fraction ; MAPK—Mitogen-Activated Protein Kinase; MARCKS—Myristoylated Alanine-Rich C-Kinase Substrate; MCP-1—Monocyte Chemoattractant Protein-1; MetS—Metabolic Syndrome; MDA—Malondialdehyde; MI—Myocardial Infarction; mitoCDN—Mitochondria-targeted Copper-Depleting Nanoparticles; MMP—Matrix Metalloproteinase; MRI—Magnetic Resonance Imaging; NADPH—Nicotinamide Adenine Dinucleotide Phosphate (reduced form); NCOA4—Nuclear Receptor Coactivator 4; NF-κB—Nuclear Factor kappa-light-chain-enhancer of activated B cells; NLRP1/NLRP3—NOD-Like Receptor Protein 1/3; NO—Nitric Oxide; NRF2—Nuclear Factor Erythroid 2–related Factor 2; NTBI—Non-Transferrin-Bound Iron; xLDL—Oxidized Low-Density Lipoprotein; OXPHOS—Oxidative Phosphorylation; PAH—Pulmonary Arterial Hypertension; Pdhx—Pyruvate Dehydrogenase Complex Component X; PDK1—3-Phosphoinositide-Dependent Protein Kinase-1; PELI1—Pellino E3 Ubiquitin Protein Ligase 1; PH—Pulmonary Hypertension; PKCα—Protein Kinase C alpha; PPAR—Peroxisome Proliferator-Activated Receptor; PUFA-PL—Polyunsaturated Fatty Acid-containing Phospholipid; PTGS2—Prostaglandin-Endoperoxide Synthase 2 (COX-2); RCD—Regulated Cell Death; RILPL2—Rab-Interacting Lysosomal Protein-Like 2; ROS—Reactive Oxygen Species; SGLT2—Sodium–Glucose Cotransporter-2; SIRT7—Sirtuin 7; SLC7A11—Solute Carrier Family 7 Member 11; SLC25A3—Mitochondrial Phosphate Carrier; SLC31A1—Gene encoding Copper Transporter 1 (CTR1); SLC40A1—Gene encoding Ferroportin; SOD—Superoxide Dismutase; GEO—Gene Expression Omnibus; STAT3—Signal Transducer and Activator of Transcription 3; STK17B—Serine/Threonine Kinase 17B; STZ—Streptozotocin; T2DM—Type 2 Diabetes Mellitus; TCA—Tricarboxylic Acid cycle; TfR1—Transferrin Receptor 1; TRAF4—TNF Receptor-Associated Factor 4; TTM—Tetrathiomolybdate; VCAM-1—Vascular Cell Adhesion Molecule-1; VEGF—Vascular Endothelial Growth Factor; VSMC—Vascular Smooth Muscle Cell; WGCNA—Weighted Gene Co-expression Network Analysis.
